# A multi-swarm greedy selection enhanced fruit fly optimization algorithm for global optimization in oil and gas production

**DOI:** 10.1371/journal.pone.0322111

**Published:** 2025-06-03

**Authors:** Yang Gao, Liang Cheng

**Affiliations:** School of Petroleum Engineering, Yangtze University, Wuhan, China; Hanshan Normal University, CHINA

## Abstract

Optimizing oil and gas production is of paramount importance in the petroleum sector, as it ensures the economic success of oil companies and meets the growing global demand for energy. The optimization of subsurface oil and gas production is critical for decision-makers, as it determines essential strategies like optimal well placement and well control parameters. Traditional reservoir production optimization methods often involve high computational costs and difficulties in achieving effective optimization. Evolutionary algorithms, inspired by biological evolution, have proven to be powerful tools for solving complex optimization challenges due to their independence from gradient information and efficient parallel processing capabilities. This paper proposes a highly efficient evolutionary algorithm for global optimization and oil and gas production optimization by enhancing the optimization performance of fruit fly optimization algorithm (FOA) through multi-swarm mechanism and greedy selection mechanism, which balance the algorithm’s search and development capabilities. Specifically, after updating the population of FOA, we first apply multi-swarm mechanism to help the population escape local optima and improve the algorithm’s search ability, and then apply greedy selection mechanism to enhance the population’s development potential. To verify the optimization performance of MGFOA, we conducted comprehensive experimental validations at IEEE CEC 2017 and IEEE CEC 2022, including ablation studies, scalability experiments, search trace visualizations, and comparisons with other similar algorithms. Finally, MGFOA significantly outperformed other comparable algorithms in oil and gas production optimization.

## 1 Introduction

Reservoir management (RM) is a critical area in the petroleum industry that has been prioritized by many oil and gas companies. According to Wiggins and Startzman [1], RM is defined as the strategic use of available technology, financial resources, and labor to optimize the economic output and recovery from a reservoir. They further detailed that RM encompasses a continuum of activities from the initial discovery of a reservoir to its eventual abandonment. In this context, production optimization is a key aspect of RM. Oil and gas firms seek to optimize hydrocarbon production not only to satisfy the increasing demand for energy but also to enhance their economic returns. One approach to increasing production is through waterflooding or water injection. Waterflooding is commonly utilized to recover additional hydrocarbons after primary recovery, which depends on natural processes such as gas cap drive and gravitational drainage [2]. Additionally, careful planning and implementation of waterflooding are crucial to avoid excessive costs during the process. As a result, waterflooding optimization has been a significant research focus for many years, assisting oil and gas companies in improving the efficiency of this technique.

Optimizing waterflooding is recognized as an engineering problem that involves the application of mathematical algorithms to determine design parameters that either maximize or minimize specific objective functions [3]. These design parameters may include well production rates, well injection rates, bottomhole pressure, and the initiation timing of waterflooding. Interestingly, the waterflooding problem can also be formulated as a multi-objective optimization problem, where more than one objective function is optimized simultaneously [4,5]. This multi-objective approach offers more practical insights for chemical and petroleum engineers, aligning closely with real-world challenges. Additionally, numerical reservoir simulation (NRS) is a commonly used tool for reservoir modeling during the field development phase and can be employed alongside other algorithms to tackle production optimization problems. However, NRS requires substantial computational resources, especially when modeling geologically complex reservoirs. This is due to NRS’s reliance on mathematical equations and physics-based models to simulate fluid flow in subsurface environments, resulting in increased computational time as the complexity of the reservoir rises. Addressing this computational demand has become a key area of research.

Traditional gradient-based methods face significant challenges and limitations when handling global optimization problems with high nonlinearity and multiple local optima [6]. Over the past few decades, numerous meta-heuristic optimization algorithms with varying attributes have been developed [7–17]. Metaheuristics are extensively utilized for addressing global optimization issues due to their simplicity, efficiency, and low computational demands. Among the more classic algorithms are: the firefly algorithm (FA) [18], moth-flame optimization algorithm (MFO) [19], particle swarm optimization (PSO) [20], bat algorithm (BA) [21], whale optimization algorithm (WOA) [22], Harris hawks optimizer (HHO) [23]. The fruit fly optimization algorithm (FOA) [24], proposed by Pan in 2012, is a recent swarm intelligence algorithm based on the visual and olfactory foraging behavior of fruit flies. However, the no free lunch (NFL) theorem indicates that no single optimizer is universally the best for all problem types [25]. Therefore, the literature includes a broad spectrum of evolutionary and swarm-based optimization algorithms. A good optimizer might achieve optimal results for a specific problem but could also converge to local optima or provide incorrect solutions for other problem classes. For example, an evolutionary and swarm-based optimizer might perform well for continuous function optimization but might fail to deliver high-quality solutions for binary feature selection problems. The performance of swarm-based optimizers can be inconsistent, sometimes failing to balance exploratory and exploitative tendencies. This does not imply that a new optimizer must be developed from scratch to achieve high-quality solutions. Instead, the NFL theorem encourages us to further refine existing optimizers for specific problem classes to address their primary shortcomings and achieve a more stable variant. Consequently, even with the availability of alternative meta-heuristics, we aim to improve the well-established FOA to achieve more stable and efficient performance.

Several investigations have been conducted on optimizing oil and gas extraction with evolutionary algorithms, for example, Wang *et al.* [26] presents an efficient and robust method for real-time production optimization under conditions of uncertainty. The production optimization problem is formulated as a Markov decision process, where a reinforcement learning agent engages with a reservoir simulator to develop a control strategy that maximizes a specified objective. To assist in the training of the intelligent agent, a population-based evolutionary algorithm is introduced to mitigate the challenges of premature convergence and insufficient exploration in reinforcement learning methods. This approach provides diverse exploratory experiences and enhances stability and robustness due to its intrinsic redundancy. Du *et al.* [27] has established an integrated optimization framework that combines Bayesian random forest (BRF) with PSO. The BRF approach is used to construct an agent model for the injection and extraction system, which is capable of accurately predicting the dynamic parameters of production wells based on injection data and production measures. This agent model is then used by the PSO algorithm to find the optimal injection configuration, utilizing Pareto frontier analysis for multi-objective optimization. Ng *et al.* [28] combines agent-based methods with particle swarm optimization for production optimization and illustrates how Long Short Term Memory (LSTM), a machine learning technique, can be applied to create agents for 3D reservoir modeling. A sampling method is used to produce a substantial number of simulation cases, which form the training database for building these agents.

Although significant studies have been performed, these algorithms frequently struggle to maintain an equilibrium between exploration and exploitation, which hampers their ability to consistently identify optimal solutions. The NFL theorem suggests that we can enhance an existing optimizer for specific problem classes to overcome its primary limitations and achieve a more stable variant. Therefore, despite the presence of other meta-heuristics, we modify the well-established FOA to achieve more consistent and efficient performance. The Multi-swarm Greedy Fruit Fly Optimization Algorithm (MGFOA) is an advanced population-based metaheuristic designed to leverage the natural foraging behavior of fruit flies for global optimization. The algorithm begins by initializing essential parameters such as the population size (*popsize*) and the maximum number of function evaluations (*MaxFEs*). Each fruit fly in the initial population is assigned random coordinates in a two-dimensional space ($X_{axis}$ and $Y_{axis}$). The initial fitness values for all individuals in the population are then computed using a predefined fitness function. The fruit fly with the minimum fitness value, denoted as bestSmell, is identified, and its corresponding index (*bestindex*) is recorded. This fruit fly’s position is used to set the $X_{axis}$ and $Y_{axis}$ for the search center, and the associated fitness value is stored as *bestCVaccuracy*. The core optimization process involves an iterative loop that continues until the number of iterations reaches Max_iterations. During each iteration, the position of each fruit fly is updated by adding a random value to the current best X and Y coordinates, simulating a random search pattern. The distance of each fruit fly from the origin is calculated, and the inverse of this distance is used to derive the sensory value ($S_{i}$). The fitness function is then applied to these sensory values to evaluate the current fitness levels. The fruit fly with the best fitness in the current iteration is identified, and if this fitness (*bestSmell*) is better than the previously recorded *bestCVaccuracy*, the new position and fitness value are updated as the current best. This process ensures that the search is continually directed towards the optimal region. The sensory values are further refined using a multi-swarm mechanism, which divides the population into subgroups to explore different regions of the search space, and a greedy selection mechanism, which favors the most promising individuals to enhance convergence speed and solution quality. In traditional Drosophila population algorithms, a single population may easily fall into local optima, while the multi-population mechanism ensures good interactions between sub-populations by dividing the population into multiple sub-populations, each of which starts independently in a different search area, and at the same time introduces an information exchange strategy. This mechanism not only enhances the diversity of the population, but also avoids premature convergence in the search process. The iterative process is repeated until the stopping criteria are met, at which point the algorithm returns the best fitness value (*bestCVaccuracy*) achieved during the search. This approach enables MGFOA to effectively balance exploration and exploitation, leading to robust performance in complex optimization problems. In summary, the main contributions of this study are outlined as follows:

1. This study introduces an enhanced version of FOA, referred to as MGFOA, which incorporates the multi-swarm mechanism and greedy selection mechanism. The two mechanisms enable MGFOA to effectively balance exploration and exploitation, resulting in robust performance in complex optimization problems.
2. The effectiveness of the proposed MGFOA was evaluated by benchmarking it against state-of-the-art evolutionary algorithms at IEEE CEC 2017 and IEEE CEC 2022. This study also offers an in-depth analysis of how the two improvement mechanisms influence the performance of MGFOA and assesses its scalability in various dimensions.
3. To assess the effectiveness of the proposed MGFOA in addressing real-world production issues, this study applies it to optimize production in three-channel reservoirs. Furthermore, the algorithm’s performance is benchmarked against other advanced evolutionary algorithms. The experimental results demonstrate the exceptional optimization capabilities of the proposed algorithm in real-world scenarios.

The structure of this study is as follows: Section 2 offers a detailed background on the FOA and related work. Section 3 discusses the two novel mechanisms embedded within the FOA. Section 4 presents a series of comparative experiments on the MGFOA. Section 5 shows the implementation of the proposed evolutionary algorithm in practical applications. Section 6 concludes the chapter and outlines potential avenues for future research.

## 2 Related work

### 2.1 Related algorithms

Many efficient evolutionary algorithms have been proposed in recent years, and some of these effective improvement strategies can greatly improve the performance of the algorithms. We will introduce these recent algorithms in this section.

Some improved evolutionary algorithms based on multiple swarm techniques have better results. Alawad *et al.* [29] proposed a new approach to PASP based on island AOA. The new algorithm is based on a structured population model called the island model. The model distributes populations of candidate solutions among islands that periodically exchange some candidate solutions with each other according to a migration protocol. Abed-alguni *et al.* [30] proposed an improved polynomial mutation-based island-based genetic algorithm (iCSPM), which adapts two improvements of the genetic algorithm. First, the island modelling strategy is introduced into the CS algorithm to enhance the ability of the CS algorithm to control population diversity. Second, the Levy flight method in CS is replaced with a highly destructive polynomial mutation method in an attempt to enhance CS exploration. Abed-alguni *et al.* [31] introduced an improved iCSPM algorithm, iCSPM with elite objection learning and multiple mutation methods (iCSPM2). iCSPM2 has three main features. First, it divides the candidate solutions into islands (subpopulations), and then distributes these islands equally among the four improved versions of CS. Abed-Alguni *et al.* [32] presents a discrete variant of the Distributed Gray Wolf Optimizer (DGWO) for scheduling dependent tasks to VMs.The scheduling process in DGWO is modelled as a minimisation problem with two objectives: computation and data transfer costs.

Some algorithms use multiple trial vector methods to improve the performance of the algorithm. Nadimi-Shahraki *et al.* [33] proposes an Improved Grey Wolf Optimizer (I-GWO).The I-GWO algorithm benefits from a new locomotor strategy, the Dimensionally Learned Hunting (DLH) based search strategy, which is inherited from the individual hunting behaviour of wolves in nature. Nadimi-Shahraki *et al.* [34] proposes an effective meta-heuristic algorithm, Multiple Trial Vector-based Differential Evolution (MTDE).MTDE features the introduction of an adaptive moving step based on the design of a new Multiple Trial Vector method, MTV, which combines different searching strategies in the form of Trial Vector Producers (TVP). strategies. Nadimi-Shahraki *et al.* [35] proposes a multiple trial vector-based sine-cosine algorithm (MTV-SCA). In MTV-SCA, a sufficient number of search strategies containing three control parameters are tuned by the multiple trial vector (MTV) method to achieve a specific goal during the search process. Nadimi-Shahraki *et al.* [36] proposes an efficient whale optimisation algorithm (EWOA-OPF) for solving optimal power flow problems. Nadimi-Shahraki *et al.* [37] proposed a discrete version of the Improved Grey Wolf Optimiser (I-GWO) algorithm, called DI-GWOCD, for efficiently detecting communities across different networks. Tabatabaei et al. [38] proposed a routing protocol technique based on clustering methods, aiming to maximize data transmission while reducing latency and energy consumption. The protocol employs a decentralized search algorithm and fuzzy logic for node clustering. To evaluate the effectiveness of the proposed method, simulations were conducted using the AFSRP protocol. Tabatabaei et al. [39] proposed an intelligent network routing method based on the symbiotic organism search algorithm. The proposed method can operate in dynamic environments and considers four criteria: available bandwidth, mobility speed, number of mobility hops, and remaining battery power. Simulation results indicate that the learning process of the symbiotic organism search algorithm significantly impacts network performance. Tabatabaei et al. [40] proposed a sensor network routing algorithm that utilizes bacterial foraging and mobile receiver algorithms to enhance energy efficiency. In the proposed method, the number of sensor nodes is determined based on the energy level on the battery surface and the distance to the forward receiver, leading to the formation of regular clusters within the network.Tabatabaei et al. [41] introduced a novel method to optimize energy consumption in wireless sensor networks for mobile target tracking applications. The proposed approach consists of two stages. In the first stage, the vortex search algorithm is used to determine the target’s path and location. In the second stage, data related to the target’s movement is transmitted between sensor nodes and the receiver, while adjusting the sleep/wake cycles of the sensor nodes.

While all of these algorithms mentioned above incorporate a variety of improvement mechanisms to enhance the performance of the algorithms, they do not take into account the global optimisation performance of the algorithms and the balance between exploitation and exploration. The Multi-swarm Greedy Fruit Fly Optimization Algorithm (MGFOA) is an advanced population-based metaheuristic designed to leverage the natural foraging behavior of fruit flies for global optimization. In traditional Drosophila population algorithms, a single population may easily fall into local optima, while the multi-population mechanism ensures good interactions between sub-populations by dividing the population into multiple sub-populations, each of which starts independently in a different search area, and at the same time introduces an information exchange strategy. This mechanism not only enhances the diversity of the population, but also avoids premature convergence in the search process. The iterative process is repeated until the stopping criteria are met, at which point the algorithm returns the best fitness value achieved during the search. This approach enables MGFOA to effectively balance exploration and exploitation, leading to robust performance in complex optimization problems. In summary, the main contributions of this study are outlined as follows:

1. This study introduces an enhanced version of FOA, referred to as MGFOA, which incorporates the multi-swarm mechanism and greedy selection mechanism. The two mechanisms enable MGFOA to effectively balance exploration and exploitation, resulting in robust performance in complex optimization problems.
2. The MGFOA is an advanced population-based metaheuristic algorithm designed to perform global optimization by leveraging the natural foraging behavior of fruit flies.
3. Multi-Group Strategy: Unlike traditional single-group approaches, MGFOA divides the population into multiple groups. This enhances exploration capabilities by allowing diverse search directions, reducing the risk of premature convergence.
4. Greedy Mechanism: MGFOA incorporates a greedy selection process to prioritize individuals with better fitness, accelerating convergence while maintaining solution quality.
5. Enhanced Global Search: By integrating both local and global search strategies within each group, MGFOA effectively balances exploration and exploitation, improving its ability to escape local optima.
6. Dynamic Interaction: The algorithm allows inter-group communication, enabling the exchange of information between groups. This dynamic interaction fosters diversity and further enhances convergence speed. These innovations make MGFOA more efficient and robust in solving complex optimization problems compared to conventional algorithms.
7. The effectiveness of the proposed MGFOA was evaluated by benchmarking it against state-of-the-art evolutionary algorithms at IEEE CEC 2017 and IEEE CEC 2022. This study also offers an in-depth analysis of how the two improvement mechanisms influence the performance of MGFOA and assesses its scalability in various dimensions.
8. To assess the effectiveness of the proposed MGFOA in addressing real-world production issues, this study applies it to optimize production in three-channel reservoirs. Furthermore, the algorithm’s performance is benchmarked against other advanced evolutionary algorithms. The experimental results demonstrate the exceptional optimization capabilities of the proposed algorithm in real-world scenarios.

### 2.2 Fruit fly optimization algorithm

**Fig 1:**
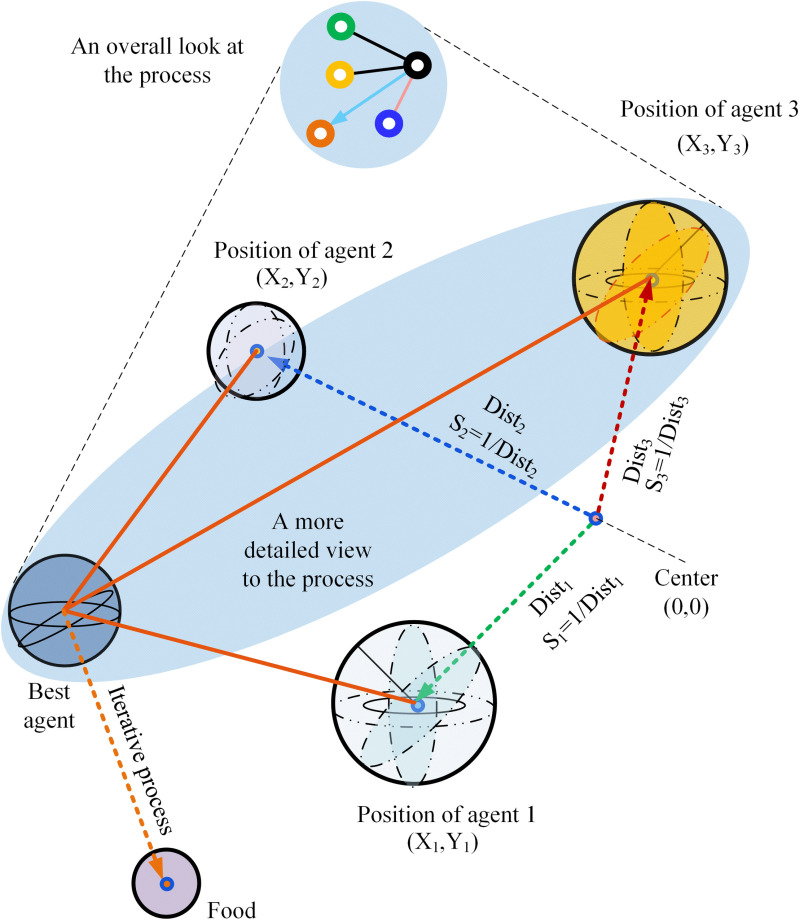
Iterative foraging process of fruit flies.

Pan *et al.* introduced the Fruit Fly Optimization Algorithm (FOA) in 2012 [24]. This artificial optimizer is inspired by the foraging behavior of fruit fly swarms. FOA seeks the optimal solution within a specific solution space by emulating the visual and olfactory mechanisms of fruit flies. The fundamental principle of FOA involves fruit flies initially flying in random directions and distances guided by their sense of smell. As they approach a food source, they switch to using their vision to locate it more accurately. After sampling the food, the fruit flies communicate to identify the location with the highest food concentration. Subsequently, the rest of the swarm moves towards this optimal location during the search process. Once this site is identified, the fruit flies continue to forage, looking for areas with even higher concentrations. The iterative foraging process of the fruit fly population is depicted in Fig 1.

The FOA algorithm can be delineated into the subsequent steps:

Step 1 Initialize parameters. Specify the maximum number of evaluations (MaxFEs), the population size (popsize), and the range of population locations (LR). Randomly define the initial position of the fruit fly population within the specified range.


$$X_{axis}=rand(LR)$$ (1)

$$Y_{axis}=rand(LR)$$ (2)

Step 2 Initialize population. The fruit fly individuals conduct random olfactory-based food searches.


$$X_{i}=X_{axis}+tempx$$ (3)

$$Y_{i}=Y_{axis}+tempy$$ (4)

tempx=rand in [−10,10] (5)

tempy=rand in [−10,10] (6)

Step 3 Population assessment. To begin with, the distance from each fruit fly individual in the population to the initial position can be evaluated using the following formula.


$${Dist}_{i}=\sqrt{X_{i}^{2}+Y_{i}^{2}}$$ (7)

Secondly, employ the reciprocal of the distance as the criterion for determining odor concentration.


$$S_{i}=1/{Dist}_{i}$$ (8)

Step 4 Calculate the fitness value. Substitute the odor concentration $S_{i}$ into the function for determining odor concentration to ascertain ${Smell}_{i}$ at the fruit fly’s location.


$${Smell}_{i}=Fitness\;function(S_{i})$$ (9)

Step 5 Find out optimal. Investigate fruit flies possessing optimal odor concentration values.


[bestSmell bestindex]=min(Smell) (10)

Step 6 Retain concentration values and coordinates. The fruit fly population maintains the optimal odor concentration value and the location information of the fruit fly while navigating to the location using vision.


$$X_{axis}=X(bestindex)$$ (11)

$$Y_{axis}=Y(bestindex)$$ (12)

Smellbest=bestSmell (13)

Step 7 Iteration process. Iterate through steps 2–5 again if the current odor concentration value is lower than that of the previous generation. Otherwise, proceed to step 6.

Here is a description of the implementation of the Fruit Fly Optimization Algorithm (FO).

**Algorithm 1. Pseudo-code of FOA**

Commence by initializing the parameters popsize and MaxFEs.

Begin by initializing the population of fruit flies with their X_axis and Y_axis coordinates.

**For**  i = 1 to popsize

    Compute the initial fitness values.

**End For**

[bestSmell bestindex]=min(Smell);

$X_{axis}=X(bestindex);$

$Y_{axis}=Y(bestindex);$

bestCVaccuarcy=bestSmell;

**While** (iteration≤Max_iteraition)

   **For** i = 1 to popsize

    $X_{i}=X_{axis}+randomValue;$

    $Y_{i}=Y_{axis}+randomValue;$

    ${Dist}_{i}=\sqrt{X_{i}^{2}+Y_{i}^{2}};$

    $S_{i}=1/{Dist}_{i};$

    $Smell(i)=Function\left(S_{i,j}\right);$

    [bestSmell bestindex]=min(Smell);

    If bestSmell<bestCVaccuarcy

        $X_{axis}=X(bestindex);$

        $Y_{axis}=Y(bestindex);$

        bestCVaccuarcy=bestSmell;

        **End If**

   **End For**

   iteration=iteration+1;

**End While**

Return bestCVaccuarcy;

## 3 Proposed MGFOA

The classical FOA stands out for its straightforward structure, limited control parameters, and user-friendly nature. Nevertheless, its convergence rate and accuracy when applied to multi-modal and complex functions are less than ideal. To mitigate these drawbacks, this paper proposes two novel mechanisms designed to enhance both the convergence speed and the solution quality of the classical FOA.

### 3.2 Multi-swarm mechanism

The topological structure of a swarm plays a crucial role in governing information dissemination among its members, thereby balancing exploration and exploitation. In this dynamic multi-swarm mechanism, the initial stage involves dividing the entire swarm into multiple equally-sized sub-swarms. This approach directs the algorithm’s search towards exploration. As evolution proceeds, the Density-based Neighborhood Search (DNS) procedure adjusts swarm dynamics: sub-swarm sizes increase while their number decreases, shifting focus towards exploitation. Moreover, the Progressive Diverse Search (PDS) strategy facilitates information sharing among sub-swarms, enhancing algorithmic exploitation capabilities. When encountering potential local optima, the Swarm Response Strategy (SRS) aids in escaping such traps, thereby bolstering exploration effectiveness. The following sections detail these innovative strategies.

During the initial stages of evolution, the swarm is partitioned into numerous sub-swarms, typically starting with each sub-swarm containing two individuals. As evolution progresses, the number of sub-swarms decreases while their sizes increase gradually. By the final stages of evolution, only one sub-swarm remains, consolidating all individuals into a unified entity. Furthermore, the effective implementation of the Density-based Neighborhood Search (DNS) strategy hinges on two critical factors: determining the initial number of sub-swarms and timing the adjustments made to these sub-swarms.

To address the primary challenge, the proposed algorithm initializes a predefined ordered set of integers $N=\left\{n_{1},n_{2},\ldots ,n_{k-1},n_{k}\right\}$. In this set, integers are arranged in descending order, denoting the number of sub-swarms. Specifically, $n_{1}>n_{2}>\ldots >n_{k-1}>n_{k}$. Each integer represents the count of sub-swarms. To facilitate the execution of the Density-based Neighborhood Search (DNS) strategy, the algorithm mandates uniform sub-swarm sizes within each iteration. Thus, the number of sub-swarms must evenly divide the total number of particles. For example, with a total of 30 particles, N={15,10, 6,5,3, 2, 1}, ensuring each element in *N* is a divisor of the total particle count. Consequently, during the initial stages of evolution, the swarm size per sub-swarm *S*_*sub*_ is set as $\frac{N}{N_{sub}}=2$. By the conclusion of the evolutionary process, *N*_*sub*_ and *S*_*sub*_ converge to 1 and 30, respectively, consolidating all particles into a single swarm.

To address the second challenge, the algorithm proposed in this paper employs a method where the adjustment period *C*_*gen*_ is set to regularly adapt the number of sub-swarms. This adjustment period is calculated as $C_{gen}=MaxGens/\left\|N\right\|$, where ‖N‖ denotes the number of elements in the integer set *N*, and MaxGens represents the maximum preset iterations before the algorithm initiates. This approach evenly divides the entire iterative process into stages corresponding to different sub-swarm numbers, ensuring a smooth transition between them. In addition to these adjustments, the Density-based Neighborhood Search (DNS) strategy incorporates a local search mechanism. This mechanism enhances the algorithm’s ability to swiftly converge towards more precise solutions by periodically applying a local search operator to the global best individual, GBEST. This operator utilizes the BFGS Quasi-Newton method on GBEST following each adjustment of the sub-swarm count.

**Fig 2:**
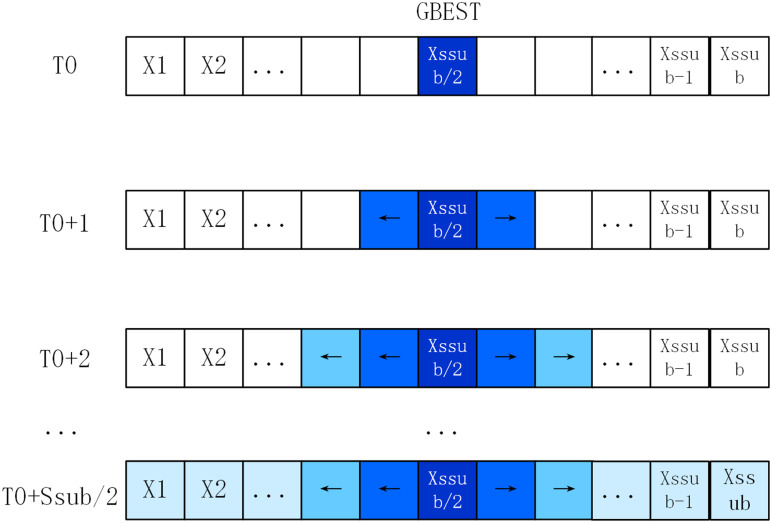
The process of information diffusion among ring topological structures.

The DNS strategy’s procedure is detailed in the subsequent pseudo-code, where $X_{j}^{i}$, $V_{j}^{i}$ and ${Pb}_{j}^{i}\;$denote the position, velocity, and historical optimal solution of the *j* th individual in the *i* th swarm, respectively. The effectiveness of the random regrouping method in our algorithm hinges on the presence or absence of stagnation, which serves as a criterion triggering a random regrouping strategy. Here, *Stag*_*best*_ denotes the number of iterations GBEST, the global optimal solution, remains stagnant. This setup enables prompt initiation of random regrouping when stagnation exceeds a predefined threshold. The determination of stagnation must consider the sub-swarm’s size; larger sub-swarms require more iterations for individuals to fully integrate valuable neighbor information. Accordingly, *Stag*_*best*_ is updated as ${Stag}_{best}=\left\lfloor Ssub/2\right\rfloor$. For instance, if the sub-swarm size is 10, *Stag*_*best*_ exceeds 5, triggering execution of the SRS strategy. Fig 2 illustrates information diffusion within a swarm, while the pseudo-code for the SRS strategy’s execution flow is detailed below.

**Fig 3:**
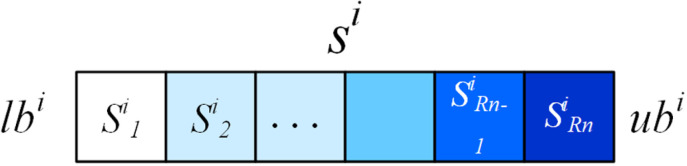
Segmentation of *i* th dimension.

In tackling complex multi-modal problems to prevent swarms from getting trapped in local optima, our approach incorporates a multi-swarm method augmented with DNS and SRS procedures, alongside a purposeful detection strategy (PDS). This strategy effectively leverages historical swarm experience to extract crucial insights and guide deliberate exploration of the most promising search spaces. A critical consideration in deploying PDS is the selection of pertinent information. In our proposed algorithm, each dimension of the search space is discretized into uniform segments, facilitating the effective execution of the PDS strategy. The segmentation of each dimension into specific segments, illustrated in Fig 3, enables the collection and utilization of individual swarm information.

As illustrated in Fig 3, *lb*^*i*^ and *ub*^*i*^ represent the lower and upper boundaries of the i-th dimension. Subsequently, the entire search space of the i-th dimension, Si, is discretized into Rn equal segments labeled as *S*^*i*^_*1*_, *S*^*i*^_*2*_,...,. Post segmentation, the frequency of occurrence of the individual historical optimal solution ${Pb}_{j}^{i}$ within each segment serves as an indicator of its exploration potential. The higher the occurrence of ${Pb}_{j}^{i}$ in a segment, the more valuable it is considered for exploration. To quantify this potential, we introduce a variable $M_{j}^{i}$, whose update formula is detailed below.


$$M_{j}^{i}=M_{j}^{i}+1\;,\;if\;{Pb}_{j}^{i}\;lies\;within\;s_{j}^{i}$$ (14)

### 3.3 Greedy selection

The decision to retain an updated individual for advancement to the next stage of evolution employs a greedy selection strategy. Specifically, this involves comparing the fitness of the updated agent with that of the primary agent. If the updated agent demonstrates superior fitness, it promptly replaces the original agent. The formula for this greedy selection strategy is articulated as follows:

In the provided updating formula, the symbol f denotes the fitness evaluation function. The formula’s description pertains to an instance of fitness minimization. Notably, if the updated fitness equals the current fitness, the subsequent evolutionary generation will incorporate the updated individuals. This mechanism is facilitated by the operator ≤ . Such design enables the swarm to navigate smoothly across flat fitness regions, thereby mitigating the risk of stagnation.

### 3.3 The proposed MGFOA

This section integrates the two mechanisms into the classical FOA framework, providing a comprehensive description of the entire MGFOA (multi-swarm mechanism and greedy selection enhanced FOA) process. The MGFOA initially implements a multi-swarm mechanism to split the population into multiple subgroups, which helps in boosting the global optimal solution search capacity. Subsequently, each subgroup independently investigates the solution space and simultaneously employs the greedy selection mechanism to enhance the algorithm’s convergence speed.

Algorithm 2 presents the pseudo-code of MGFOA. The Multi-swarm Greedy Fruit Fly Optimization Algorithm (MGFOA) is an advanced population-based metaheuristic designed to leverage the natural foraging behavior of fruit flies for global optimization. The algorithm begins by initializing essential parameters such as the population size (*popsize*) and the maximum number of function evaluations (*MaxFEs*). Each fruit fly in the initial population is assigned random coordinates in a two-dimensional space ($X_{axis}$ and $Y_{axis}$). The initial fitness values for all individuals in the population are then computed using a predefined fitness function. The fruit fly with the minimum fitness value, denoted as bestSmell, is identified, and its corresponding index (*bestindex*) is recorded. This fruit fly’s position is used to set the $X_{axis}$ and $Y_{axis}$ for the search center, and the associated fitness value is stored as *bestCVaccuracy*. The core optimization process involves an iterative loop that continues until the number of iterations reaches Max_iterations. During each iteration, the position of each fruit fly is updated by adding a random value to the current best X and Y coordinates, simulating a random search pattern. The distance of each fruit fly from the origin is calculated, and the inverse of this distance is used to derive the sensory value ($S_{i}$). The fitness function is then applied to these sensory values to evaluate the current fitness levels. The fruit fly with the best fitness in the current iteration is identified, and if this fitness (*bestSmell*) is better than the previously recorded *bestCVaccuracy*, the new position and fitness value are updated as the current best. This process ensures that the search is continually directed towards the optimal region. The sensory values are further refined using a multi-swarm mechanism, which divides the population into subgroups to explore different regions of the search space, and a greedy selection mechanism, which favors the most promising individuals to enhance convergence speed and solution quality. In traditional Drosophila population algorithms, a single population may easily fall into local optima, while the multi-population mechanism ensures good interactions between sub-populations by dividing the population into multiple sub-populations, each of which starts independently in a different search area, and at the same time introduces an information exchange strategy. This mechanism not only enhances the diversity of the population, but also avoids premature convergence in the search process. The iterative process is repeated until the stopping criteria are met, at which point the algorithm returns the best fitness value (*bestCVaccuracy*) achieved during the search. This approach enables MGFOA to effectively balance exploration and exploitation, leading to robust performance in complex optimization problems.

The computational complexity of MOFOA depends on factors such as the population size (popsize), dimensionality (dim), number of subgroups (M), and the maximum number of evaluations (MaxFEs). The iteration count T is calculated as T = MaxFEs/N, where N represents the number of evaluations required for a single execution of the algorithm. In the proposed method, the time complexity analysis primarily involves three key steps: initialization, fitness estimation of fruit flies, and the implementation of the outpost mechanism. Specifically, the complexities are as follows: O(Initialization) = O(M×popsize×dim + N × logN), O(Fitness Estimation)=O(T × M×(popsize×dim)) +N × logN. Combining these, the overall complexity is expressed as O(MGFOA) = O(M×popsize×dim + N × logN)+O(T × M×(popsize×dim + N)). It is worth noting that the time complexity of MGFOA is similar to that of FOA.MGFOA does not increase the time complexity.

**Algorithm 2. Pseudo-code of MGFOA**

Commence by initializing the parameters popsize and MaxFEs.

Begin by initializing the population of fruit flies with their X_axis and Y_axis coordinates.

**For**  i = 1 to popsize

   Compute the initial fitness values.

**End For**

[bestSmell bestindex]=min(Smell); % bestSmell: minimum value. bestindex: indicator.

$X_{axis}=X(bestindex);$

$Y_{axis}=Y(bestindex);$

bestCVaccuarcy=bestSmell; % bestCVaccuarcy:Optimal fitness value.

**While (** iteration≤Max_iteraition)

   **For** i = 1 to popsize

        $X_{i}=X_{axis}+randomValue;$ % randomValue: random value

        $Y_{i}=Y_{axis}+randomValue;$

        ${Dist}_{i}=\sqrt{X_{i}^{2}+Y_{i}^{2}};$

        $S_{i}=1/{Dist}_{i};$

        $Smell(i)=Function\left(S_{i,j}\right);$ % Smell: population

        [bestSmell bestindex]=min(Smell);

        If bestSmell<bestCVaccuarcy

          $X_{axis}=X(bestindex);$

          $Y_{axis}=Y(bestindex);$

          bestCVaccuarcy=bestSmell;

       **End If**

   **End** For

   Updating $S_{i,j}$ by multi-swarm mechanism;

   Updating $S_{i,j}$ by greedy selection mechanism;

   iteration=iteration+1;

**End While**

**Return** bestCVaccuarcy;

## 4 Experiments and results

This section presents a comprehensive evaluation of the proposed algorithm’s performance through numerous experiments. These include ablation studies, extension tests, historical search trace reviews, comparative assessments with other algorithms, and the application of the COFOA method to real-world engineering challenges. Ablation experiments were performed to exemplify the facilitation of two mechanisms in MGFOA. To demonstrate that each mechanism contributes to the improvement of FOA performance. The history search experiment is designed to reflect the global optimisation capability of the algorithm, to demonstrate the different focus of the algorithm in the pre-search and post-search phases, and to reflect the balance of the algorithm’s search and development capabilities. Comparison experiments with other algorithms are conducted to demonstrate the excellent optimisation performance of the proposed algorithm.

### 4.1 Benchmark functions

#### 4.1.1 IEEE CEC 2017 benchmark functions.

Table 1 shows the details of the IEEE CEC 2017 benchmark functions.

**Table 1:** IEEE CEC 2017 benchmark function specifications.

Function Equation	Dim	Optimum
$f_{1}(x)=x_{1}^{2}+10^{6}\sum_{i=2}^{D}x_{i}^{2}$	30	100
$f_{2}(x)=\sum_{i=1}^{D}\left|x_{i}^{2}\right|$	30	200
$f_{3}(x)=\sum_{i=1}^{D}x_{i}^{2}+{\left(\sum_{i=1}^{D}0.5x_{i}^{2}\right)}^{2}+{\left(\sum_{i=1}^{D}0.5x_{i}^{2}\right)}^{4}$	30	300
$f_{4}(x)=\sum_{i=1}^{D-1}\left(100{\left(x_{i}^{2}-x_{i+1}\right)}^{2}+{\left(x_{i}-1\right)}^{2}\right)$	30	400
$f_{5}(x)=\sum_{i=1}^{D}\left(x_{i}^{2}-10\cos\left(2\pi x_{i}\right)+10\right)$	30	500
$f_{6}(x)=g\left(x_{1},x_{2}\right)+g\left(x_{2},x_{3}\right)+\ldots +g\left(x_{\text{D-}1},x_{D}\right)+g\left(x_{D},x_{1}\right)$ $g(x,y)=0.5+\frac{\left(\sin^{2}\left(\sqrt{x^{2}+y^{2}}\right)-0.5\right)}{{\left(1+0.001\left(x^{2}+y^{2}\right)\right)}^{2}}$	30	600
	30	700
$f_{8}(x)=\sum_{i=1}^{D}\left(z_{i}^{2}-10\cos\left(2\pi z_{i}\right)+10\right)+{f_{13}}^{*}$	30	800
$f_{9}(x)=\sin^{2}\left(\pi w_{1}\right)+\sum_{i=1}^{D}{\left(w_{i}-1\right)}^{2}\left[1+10\sin^{2}\left(\pi w_{i}+1\right)\right]+{\left(w_{D}-1\right)}^{2}\left[1+\sin^{2}\left(2\pi w_{D}\right)\right]$	30	900
$f_{10}(x)=418.9829\times D-\sum_{i=1}^{D}g\left(z_{i}\right)\;,\;z_{i}=x_{i}+4.209687462275036e+002$	30	1000
$f_{11}(x)={\sum_{i=1}^{D}\left(10^{6}\right)}^{\frac{i-1}{D-1}}x_{i}^{2}$	3	1100
$f_{12}(x)=10^{6}x_{1}^{2}+\sum_{i=2}^{D}x_{i}^{2}$	3	1200
$f_{13}(x)=-20\exp\left(-0.2\sqrt{\frac{1}{D}\sum_{i=1}^{D}x_{i}^{2}}\right)-\exp\left(\frac{1}{D}\sum_{i=1}^{D}\cos\left(2\pi x_{i}\right)\right)+20+e$	3	1300
$f_{14}(x)=\sum_{i=1}^{D}\left(\sum_{k=0}^{kmax}\left[a^{k}\cos\left(2\pi b^{k}(x+0.5\right))\right]\right)-D\sum_{k=0}^{kmax}\left[a^{k}\cos\left(2\pi b^{k}\mathrm{.0}\mathrm{.5}\right)\right]$	4	1400
$f_{15}(x)=\sum_{i=1}^{D}\frac{x_{i}^{2}}{4000}-\prod_{i=1}^{D}\cos\left(\frac{x_{i}}{\sqrt{i}}\right)+1$	4	1500
$f_{16}(x)=\frac{10}{D^{2}}{\prod_{i=1}^{D}\left(1+i\sum_{j=1}^{32}\frac{\left|2^{j}x_{i}-round\left(2^{j}x_{i}\right)\right|}{2^{j}}\right)}^{\frac{10}{D^{1.2}}}-\frac{10}{D^{2}}$	4	1600
$f_{17}(x)={\left|\sum_{i=1}^{D}x_{i}^{2}-D\right|}^{1/4}+\left(0.5\sum_{i=1}^{D}x_{i}^{2}+\sum_{i=1}^{D}x_{i}\right)/D+0.5$	5	1700
$f_{18}(x)={\left|{\left(\sum_{i=1}^{D}x_{i}^{2}\right)}^{2}-{\left(\sum_{i=1}^{D}x_{i}\right)}^{2}\right|}^{1/4}+\left(0.5\sum_{i=1}^{D}x_{i}^{2}+\sum_{i=1}^{D}x_{i}\right)/D+0.5$	5	1800
$f_{19}(x)=f_{7}\left(f_{4}\left(x_{1},x_{2}\right)\right)+f_{7}\left(f_{4}\left(x_{2},x_{3}\right)\right)+\ldots +f_{7}\left(f_{4}\left(x_{D-1},x_{D}\right)\right)+f_{7}\left(f_{4}\left(x_{D},x_{1}\right)\right)$	5	1900
$f_{20}(x)={\left[\frac{1}{D-1}\sum_{i=1}^{D-1}\left(\sqrt{s_{i}}\cdot \left(\sin\left(50.0s_{i}^{0.2}\right)+1\right)\right)\right]}^{2},s_{i}=\sqrt{x_{i}^{2}+x_{i+1}^{2}}$	6	2000
$f_{21}(x)=f_{1}\left(M\left(x-o_{1}\right)\right)+{f_{21}}^{*}$	3	2100
$f_{22}(x)=f_{2}\left(M\left(x-o_{2}\right)\right)+{f_{22}}^{*}$	3	2200
$f_{23}(x)=f_{3}\left(M\left(x-o_{3}\right)\right)+{f_{23}}^{*}$	4	2300
$f_{24}(x)=f_{4}\left(M\left(\frac{2.048\left(x-o_{4}\right)}{100}\right)+1\right)+{f_{24}}^{*}$	4	2400
$f_{25}(x)=f_{5}\left(M\left(x-o_{5}\right)\right)+{f_{25}}^{*}$	5	2500
$f_{26}(x)=f_{20}\left(M\left(\frac{2.048\left(x-o_{6}\right)}{100}\right)\right)+{f_{26}}^{*}$	5	2600
$f_{27}(x)=f_{7}\left(M\left(\frac{600\left(x-o_{7}\right)}{100}\right)\right)+{f_{27}}^{*}$	6	2700
$f_{28}(x)=f_{8}\left(\frac{5.12\left(x-o_{8}\right)}{100}\right)+{f_{28}}^{*}$	6	2800
$f_{29}(x)=f_{9}\left(M\left(\frac{5.12\left(x-o_{9}\right)}{100}\right)\right)+{f_{29}}^{*}$	3	2900
$f_{30}(x)=f_{30}\left(M\left(\frac{1000\left(x-o_{10}\right)}{100}\right)\right)+{f_{30}}^{*}$	3	3000

#### 4.1.2 IEEE CEC 2022 benchmark functions.

Table 2 shows the details of the IEEE CEC 2022 benchmark functions.

**Table 2:** IEEE CEC 2022 benchmark function specifications.

Functions	Describe	$f_{i}$
F1	Shifted and full Rotated Zakharov	300
F2	Shifted and full Rotated Rosenbrock	400
F3	Shifted and full Rotated Expanded Schaffer’s f6	600
F4	Shifted and full Rotated Non-Continuous Restrain	800
F5	Shifted and full Rotated Levy	900
F6	Hybrid	1800
F7	Hybrid	2000
F8	Hybrid	2200
F9	Composition	2300
F10	Composition	2400
F11	Composition	2600
F12	Composition	2700

### 4.2 Ablation analysis

This section explores the improved effects of two enhancement mechanisms on MFFOA through ablative experiments, which are essential in scientific research. Ablative experiments are crucial for validating the robustness and reliability of research findings. By systematically removing a variable or factor and observing its impact, these experiments help confirm the presence of observed effects and eliminate alternative explanations. This approach allows researchers to understand the contribution and importance of each factor in the study, thus confirming the reliability of the results and reducing potential confounding variables. Ablative experiments are indispensable for verifying scientific hypotheses, supporting research conclusions, and improving the credibility and reproducibility of research. Table 3 presents the experimental results, where MFOA represents FOA improved solely by the multi-swarm mechanism, and GFOA indicates FOA improved exclusively by the greedy selection mechanism. Conducting 30 independent experiments on CEC 2017 benchmark functions, the data show that MGFOA, enhanced by both mechanisms, has a significant advantage over FOA improved by either mechanism alone. Specifically, MGFOA outperforms MFOA in 8 functions and GFOA in 18 functions, demonstrating that the combined use of both mechanisms greatly enhances FOA, something that cannot be achieved by either mechanism alone.

**Table 3:** Ablation analysis.

Algorithm	Rank	+/ = /-	AVG
MGFOA	1	~	1.8
MFOA	2	8/3/19	2.8
GFOA	3	18/5/7	2.5
FOA	4	15/3/12	2.9

### 4.3 Scalability analysis

The scalability of MGFOA is evaluated in this section through tests with different dimensions. Assessing scalability is critical to understanding the performance of evolutionary computing algorithms on large-scale issues. By modifying problem sizes across various dimensions, the algorithm’s competence in handling different sizes and complexities can be evaluated. These experiments measure the algorithm’s resource use, time efficiency, and solution quality to establish its suitability and limitations. Scalability tests are essential for delivering reliable solutions for extensive problems in real-world applications, enhancing the application of evolutionary computing. This study considers three dimensions: 30, 50, and 100, which are standard benchmarks in evolutionary computing, illustrating MGFOA’s global optimization capabilities. Table 4 presents the scalability test outcomes, indicating that MGFOA consistently outperforms FOA in all dimensions. The original FOA serves as a comparison benchmark, demonstrating MGFOA’s superiority in different dimensions.

**Table 4:** Scalability tests in three dimensions.

	Dim	30		50		100	
	Metric	MGFOA	FOA	MGFOA	FOA	MGFOA	FOA
**F1**	AVG	4.26E + 03	5.14E + 03	5.95E + 03	6.87E + 03	7.21E + 03	1.07E + 04
	STD	1.04E + 06	5.80E + 05	2.36E + 06	7.81E + 05	5.07E + 06	1.35E + 06
**F2**	AVG	1.05E + 07	5.01E + 07	7.21E + 27	3.94E + 28	7.16E + 77	3.92E + 78
	STD	6.85E + 15	3.70E + 16	8.45E + 30	4.63E + 31	1.15E + 98	6.31E + 98
**F3**	AVG	4.85E + 03	2.57E + 03	4.92E + 04	1.25E + 04	2.40E + 05	3.32E + 04
	STD	1.89E + 03	3.42E + 03	1.03E + 04	7.91E + 03	8.04E + 04	2.08E + 04
**F4**	AVG	4.76E + 02	2.37E + 01	5.05E + 02	5.41E + 01	6.41E + 02	2.91E + 01
	STD	4.95E + 02	3.13E + 01	5.59E + 02	4.93E + 01	6.53E + 02	3.44E + 01
**F5**	AVG	5.46E + 02	2.70E + 01	6.40E + 02	9.21E + 01	1.21E + 03	1.36E + 02
	STD	5.36E + 02	1.12E + 01	5.76E + 02	3.51E + 01	1.13E + 03	6.26E + 01
**F6**	AVG	6.00E + 02	1.52E-02	6.00E + 02	9.19E-03	6.00E + 02	1.17E-02
	STD	6.00E + 02	3.16E-02	6.00E + 02	2.10E-01	6.00E + 02	6.83E-02
**F7**	AVG	7.93E + 02	3.26E + 01	1.01E + 03	7.56E + 01	1.55E + 03	1.32E + 02
	STD	7.74E + 02	2.46E + 01	9.29E + 02	9.44E + 01	1.49E + 03	7.74E + 01
**F8**	AVG	8.42E + 02	1.02E + 01	9.34E + 02	8.00E + 01	1.50E + 03	1.55E + 02
	STD	8.36E + 02	9.34E + 00	9.13E + 02	7.35E + 01	1.40E + 03	1.32E + 02
**F9**	AVG	9.04E + 02	5.08E + 00	9.06E + 02	5.21E + 00	9.09E + 02	5.38E + 00
	STD	9.36E + 02	1.45E + 02	9.80E + 02	8.32E + 01	1.09E + 03	9.25E + 01
**F10**	AVG	4.47E + 03	1.55E + 03	9.19E + 03	2.73E + 03	2.61E + 04	5.68E + 03
	STD	3.19E + 03	5.44E + 02	6.29E + 03	2.46E + 03	2.36E + 04	2.04E + 03
**F11**	AVG	1.13E + 03	2.60E + 01	1.16E + 03	2.74E + 01	1.64E + 03	1.84E + 02
	STD	1.45E + 03	2.72E + 02	1.23E + 03	9.66E + 01	2.43E + 03	9.85E + 02
**F12**	AVG	2.10E + 05	2.19E + 05	1.76E + 06	1.02E + 06	2.69E + 06	1.08E + 06
	STD	2.95E + 06	2.10E + 06	9.99E + 06	3.73E + 06	2.80E + 07	1.14E + 07
**F13**	AVG	1.80E + 04	2.04E + 04	6.90E + 03	7.44E + 03	5.45E + 03	4.58E + 03
	STD	1.34E + 06	1.49E + 06	2.38E + 06	1.10E + 06	4.03E + 05	2.69E + 05
**F14**	AVG	4.50E + 04	3.81E + 04	8.64E + 04	6.27E + 04	2.70E + 05	9.63E + 04
	STD	7.32E + 05	1.08E + 06	2.31E + 06	1.79E + 06	3.39E + 06	1.72E + 06
**F15**	AVG	5.89E + 03	5.08E + 03	9.93E + 03	6.49E + 03	4.12E + 03	3.62E + 03
	STD	4.54E + 04	7.94E + 04	2.17E + 05	2.67E + 05	2.79E + 05	3.05E + 05
**F16**	AVG	2.44E + 03	2.75E + 02	2.87E + 03	4.11E + 02	6.14E + 03	2.02E + 03
	STD	2.37E + 03	2.47E + 02	2.73E + 03	3.79E + 02	4.93E + 03	1.84E + 03
**F17**	AVG	2.03E + 03	2.10E + 02	2.60E + 03	3.09E + 02	5.31E + 03	9.18E + 02
	STD	2.09E + 03	1.93E + 02	2.41E + 03	2.92E + 02	4.36E + 03	1.11E + 03
**F18**	AVG	2.35E + 05	2.30E + 05	1.27E + 06	1.18E + 06	2.79E + 06	1.54E + 06
	STD	1.35E + 06	1.56E + 06	3.86E + 06	6.13E + 06	3.21E + 06	1.98E + 06
**F19**	AVG	1.44E + 04	1.53E + 04	1.59E + 04	1.10E + 04	5.15E + 03	2.85E + 03
	STD	7.63E + 04	1.36E + 05	8.54E + 04	1.07E + 05	3.11E + 05	2.17E + 05
**F20**	AVG	2.28E + 03	1.56E + 02	2.73E + 03	2.79E + 02	5.62E + 03	1.01E + 03
	STD	2.37E + 03	2.18E + 02	2.52E + 03	2.39E + 02	4.54E + 03	9.98E + 02
**F21**	AVG	2.34E + 03	1.60E + 01	2.46E + 03	8.72E + 01	3.02E + 03	1.21E + 02
	STD	2.34E + 03	8.55E + 00	2.40E + 03	6.93E + 01	2.98E + 03	4.44E + 01
**F22**	AVG	2.30E + 03	1.23E + 00	1.04E + 04	3.28E + 03	2.81E + 04	9.27E + 03
	STD	2.78E + 03	9.30E + 02	8.63E + 03	3.29E + 03	2.39E + 04	6.22E + 03
**F23**	AVG	2.71E + 03	1.41E + 01	2.83E + 03	2.13E + 01	3.03E + 03	3.21E + 01
	STD	2.71E + 03	1.15E + 01	2.82E + 03	3.02E + 01	3.06E + 03	4.69E + 01
**F24**	AVG	2.88E + 03	2.54E + 01	3.07E + 03	1.01E + 02	3.56E + 03	6.69E + 01
	STD	2.87E + 03	1.71E + 01	3.10E + 03	8.04E + 01	3.61E + 03	5.65E + 01
**F25**	AVG	2.89E + 03	6.88E + 00	3.02E + 03	3.63E + 01	3.24E + 03	5.97E + 01
	STD	2.90E + 03	1.92E + 01	3.05E + 03	3.18E + 01	3.27E + 03	4.96E + 01
**F26**	AVG	3.89E + 03	4.95E + 02	4.83E + 03	4.63E + 02	9.67E + 03	2.09E + 03
	STD	4.06E + 03	3.65E + 02	4.60E + 03	6.39E + 02	8.37E + 03	6.67E + 02
**F27**	AVG	3.23E + 03	1.57E + 01	3.39E + 03	7.04E + 01	3.48E + 03	6.10E + 01
	STD	3.24E + 03	1.51E + 01	3.46E + 03	1.02E + 02	3.55E + 03	6.83E + 01
**F28**	AVG	3.20E + 03	5.14E + 01	3.29E + 03	2.15E + 01	3.36E + 03	2.45E + 01
	STD	3.23E + 03	2.42E + 01	3.31E + 03	2.25E + 01	3.42E + 03	3.53E + 01
**F29**	AVG	3.69E + 03	1.92E + 02	3.84E + 03	3.52E + 02	5.50E + 03	5.38E + 02
	STD	3.67E + 03	2.34E + 02	3.84E + 03	2.57E + 02	4.97E + 03	3.29E + 02
**F30**	AVG	9.17E + 03	4.95E + 03	1.07E + 06	3.36E + 05	1.26E + 04	6.41E + 03
	STD	1.62E + 05	1.27E + 05	1.23E + 06	3.45E + 05	1.25E + 06	6.60E + 05
**+/-/=**	~	~	17/6/7	~	14/8/8	~	15/10/5

**Fig 4:**
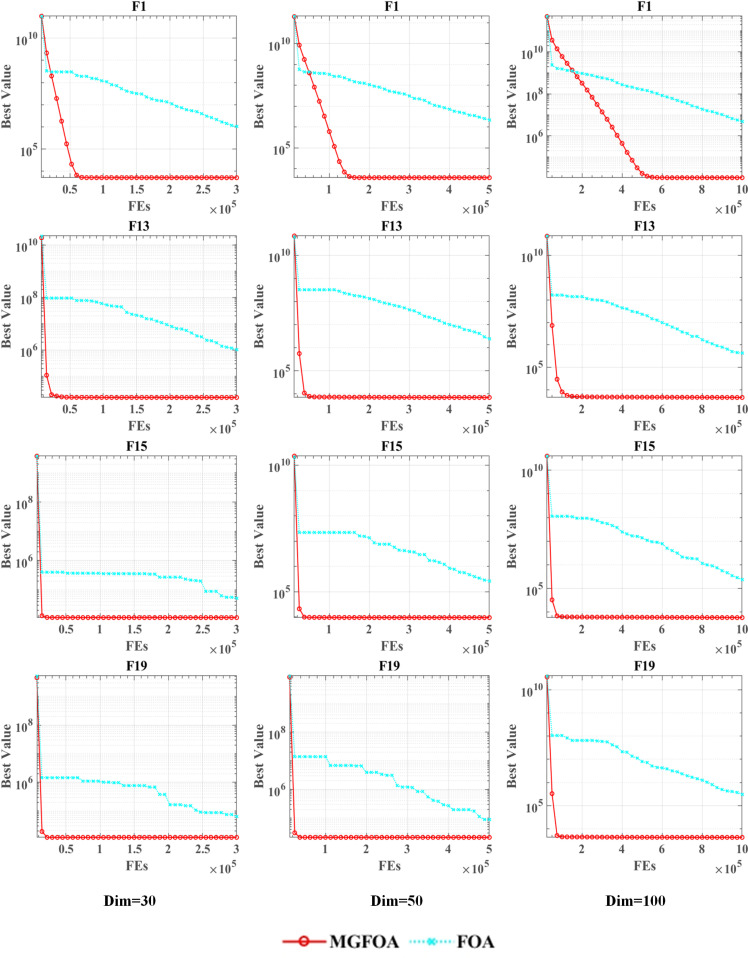
Scalability analysis on the IEEE CEC 2017 benchmark functions.

In Fig 4, the convergence curves of MGFOA (in red) and FOA (in blue) are presented for selected test functions. The dimensions analyzed are 30, 50, and 100, and the chosen test functions are F1, F13, F15, and F19 from the CEC 2017 benchmarks. The figure highlights that MGFOA exhibits superior convergence speed and accuracy compared to FOA.

### 4.4 Historical searches

**Fig 5:**
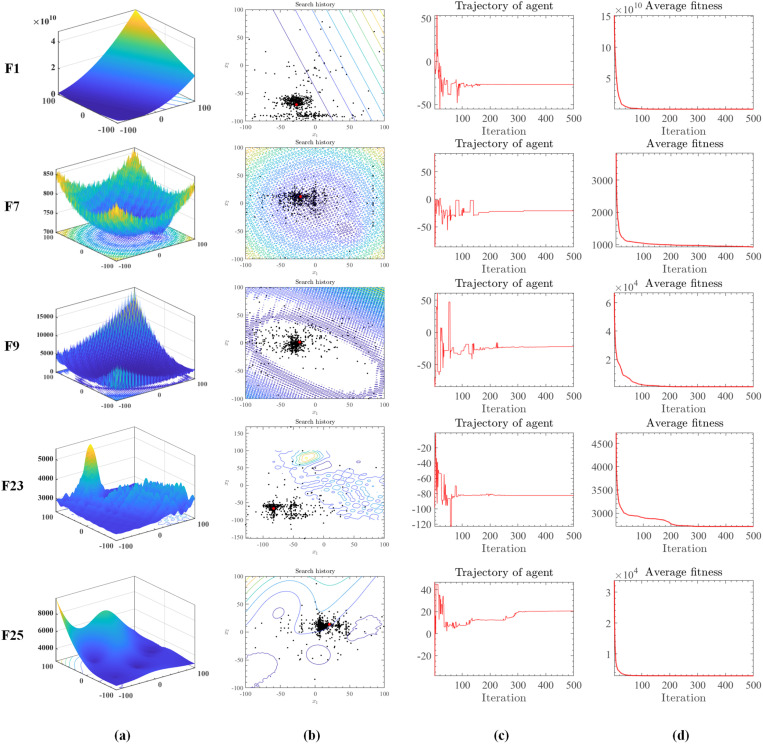
Evolutionary trajectory of MGFOA on the IEEE CEC 2017 benchmark functions.

The significance of visualizing algorithmic search processes in evolutionary computing research is paramount. Visual tools enable researchers to intuitively monitor the algorithm’s search trajectory within the solution space, its speed, and its ability to avoid local optima. This helps in understanding the algorithm’s operational principles and behavior, offering valuable insights for optimization. Visual experiments also help identify algorithmic limitations and potential issues, leading to further improvements. Thus, visual experiments of algorithmic search processes are essential for the comprehensive investigation and refinement of evolutionary computing algorithms, enhancing their development and practical application. To provide a clear understanding of MGFOA’s search process, Fig 5 illustrates its historical trajectory on IEEE CEC 2017 benchmark functions, including F1, F7, F9, F23, and F25. Fig 5a shows simulated images of these functions, while Fig 5b details the historical search path of MGFOA. Red points represent global optima, and black points indicate the optimizer’s findings at each iteration. MGFOA’s trajectory exhibits a marked inclination towards optimal values, avoiding local optima. The black dots surrounding the red dot and those evenly spread across the search space highlight MGFOA’s global exploration ability. Fig 5c illustrates the relative discrepancy from the optimal value at each iteration, with MGFOA stabilizing around 500 iterations. Lastly, Fig 5d depicts the average fitness values at the end of each iteration, showing a general downward trend.

### 4.5 Comparison of other related algorithms

#### 4.5.1 Comparative experiments at CEC 2017 benchmark functions.

This section evaluates MGFOA using the IEEE CEC 2017 benchmark functions. The Wilcoxon signed-rank test [42] and Friedman test [43] was employed to evaluate performance. For fair comparison, all algorithms were tested under uniform conditions. The population size (popsize) was set to 30, with 3 sub-populations, and the maximum evaluation number (MaxFEs) was uniformly set to 300,000. Each algorithm was executed 30 times on the benchmark functions to minimize the impact of randomness. The Friedman test [52] was used to evaluate the performance of all algorithms on the benchmark functions, providing rankings based on average performance. This test ranks the average performance of all algorithms for further statistical analysis and reports the average ranking values. To highlight the results, the best outcomes for each function in the table are bolded.

Table 5 presents the results of a comprehensive comparative study evaluating the performance of the MGFOA against a suite of alternative algorithms on the IEEE CEC 2017 benchmark functions. The competing algorithms involved in this experiment include HGWO [44], WEMFO [45], mSCA [46], SCADE [47], CCMWOA [48], QCSCA [49], BWOA [50], CCMSCSA [51], CLACO [52], BLPSO [53], GCHHO [54]. The experiments were conducted over 30 runs for each benchmark function, and the results are reported in terms of algorithm rank, the number of wins/draws/losses (denoted as + / = /-), and the average performance score (AVG). MGFOA demonstrates a commanding lead in performance, securing the top rank with a notably low average score of 1.88E + 00. The comparative column is marked as “~,” indicating that MGFOA is used as the reference algorithm in this analysis. The data highlights MGFOA’s superior optimization capabilities, consistently outperforming the other competing algorithms across the benchmarks. This result underscores the robustness and effectiveness of MGFOA in handling complex optimization tasks. In contrast, HGWO, which ranks 9th, exhibits significantly lower performance with an average score of 9.32E + 00. The “+/=/-” value of 30/0/0 illustrates that MGFOA outperforms HGWO in every comparison across the benchmark functions, indicating that HGWO is unable to match the optimization efficiency of MGFOA. Similarly, SCADE and CCMWOA, ranked 11th and 12th respectively, show consistent underperformance with no wins against MGFOA and average scores of 1.06E + 01 and 1.13E + 01. This underscores their relative inadequacy in comparison to MGFOA. The algorithms WEMFO and mSCA, ranked 6th and 7th respectively, show moderate competitiveness with average scores of 5.77E + 00 and 6.23E + 00. Both manage to achieve 5 wins against MGFOA, indicating their potential to occasionally outperform MGFOA. However, the majority of the results still favor MGFOA, affirming its overall superiority in optimization performance. CLACO, which ranks 2nd with an average score of 2.54E + 00, demonstrates relatively strong performance but is still outperformed by MGFOA, as evidenced by the “+/=/-” metric of 17/8/5. This suggests that while CLACO is competitive, it does not consistently surpass MGFOA. Similarly, QCSCA and BLPSO, ranked 3rd and 4th respectively, with average scores of 3.50E + 00 and 3.76E + 00, show strong but not leading performance. Their “+/=/-” metrics of 19/4/7 and 21/1/8 highlight their occasional superiority but overall inferiority to MGFOA.

**Table 5:** Experiments comparing MGFOA with alternative competing algorithms on the IEEE CEC 2017 benchmark functions.

Algorithm	Rank	+/ = /-	AVG
MGFOA	1	~	1.88E + 00
HGWO	9	30/0/0	9.32E + 00
WEMFO	6	25/0/5	5.77E + 00
mSCA	7	25/0/5	6.23E + 00
SCADE	11	30/0/0	1.06E + 01
CLACO	2	17/8/5	2.54E + 00
BLPSO	4	21/1/8	3.76E + 00
CCMWOA	12	30/0/0	1.13E + 01
QCSCA	3	19/4/7	3.50E + 00
BWOA	10	30/0/0	9.67E + 00
CCMSCSA	8	29/0/1	8.21E + 00
GCHHO	5	20/2/8	5.26E + 00

The algorithms BWOA and CCMSCSA, ranked 10th and 8th respectively, exhibit underwhelming performance with average scores of 9.67E + 00 and 8.21E + 00, and fail to pose any significant challenge to MGFOA. Finally, GCHHO, ranked 5th with an average score of 5.26E + 00, performs relatively well but is still consistently outperformed by MGFOA, reflecting the latter’s superior optimization capability. In conclusion, the experimental results clearly indicate that MGFOA outperforms all other competing algorithms on the IEEE CEC 2017 benchmark functions. MGFOA’s top ranking and the lowest average performance score highlight its effectiveness and robustness in tackling complex optimization problems, affirming its potential as a leading optimization framework in various applications. Tables 6 and 7 demonstrates the p-values of the comparison experiments. In traditional Drosophila population algorithms, a single population may easily fall into local optima, while the multi-population mechanism ensures good interactions between sub-populations by dividing the population into multiple sub-populations, each of which starts independently in a different search area, and at the same time introduces an information exchange strategy. This mechanism not only enhances the diversity of the population, but also avoids premature convergence in the search process.

**Table 6:** The p-values algorithm.

Function	MGFOA	HGWO	WEMFO	mSCA	SCADE	CLACO	BLPSO
**F1**	N/A	1.73440E-06	8.91873E-05	1.73440E-06	1.73440E-06	1.73440E-06	1.73440E-06
**F2**	N/A	1.73440E-06	1.73440E-06	1.73440E-06	1.73440E-06	1.73440E-06	1.73440E-06
**F3**	N/A	1.73440E-06	6.56411E-02	1.73440E-06	1.73440E-06	1.73440E-06	1.73440E-06
**F4**	N/A	1.73440E-06	1.65655E-02	1.73440E-06	1.73440E-06	2.87860E-06	1.73440E-06
**F5**	N/A	1.73440E-06	1.92092E-06	1.73440E-06	1.73440E-06	1.73440E-06	1.73440E-06
**F6**	N/A	1.73440E-06	1.73440E-06	1.73440E-06	1.73440E-06	1.73440E-06	1.73440E-06
**F7**	N/A	1.73440E-06	2.60333E-06	1.73440E-06	1.73440E-06	1.73440E-06	1.73440E-06
**F8**	N/A	1.73440E-06	1.73440E-06	1.73440E-06	1.73440E-06	1.73440E-06	1.73440E-06
**F9**	N/A	1.73440E-06	1.73440E-06	1.73440E-06	1.73440E-06	1.73440E-06	1.73440E-06
**F10**	N/A	1.97295E-05	3.70935E-01	1.23808E-05	1.73440E-06	3.40526E-05	2.89477E-01
**F11**	N/A	1.73440E-06	1.73440E-06	1.73440E-06	1.73440E-06	1.73440E-06	1.73440E-06
**F12**	N/A	1.73440E-06	3.18168E-06	1.73440E-06	1.73440E-06	1.73440E-06	1.73440E-06
**F13**	N/A	1.73440E-06	3.18168E-06	1.73440E-06	1.73440E-06	3.50090E-02	1.73440E-06
**F14**	N/A	1.73440E-06	7.81264E-01	1.73440E-06	1.73440E-06	6.03501E-03	9.26255E-01
**F15**	N/A	1.73440E-06	7.69086E-06	1.73440E-06	1.73440E-06	1.05695E-04	2.35342E-06
**F16**	N/A	1.73440E-06	3.60943E-03	1.73440E-06	1.73440E-06	9.31566E-06	1.98610E-01
**F17**	N/A	3.51524E-06	6.26828E-02	2.35342E-06	2.35342E-06	8.46608E-06	9.42611E-01
**F18**	N/A	2.60333E-06	1.60464E-04	1.73440E-06	1.73440E-06	4.07151E-05	3.11232E-05
**F19**	N/A	1.73440E-06	3.31726E-04	1.73440E-06	1.73440E-06	1.19734E-03	1.73440E-06
**F20**	N/A	7.69086E-06	7.73094E-03	4.72920E-06	1.73440E-06	3.88218E-06	2.71155E-01
**F21**	N/A	1.73440E-06	1.73440E-06	1.73440E-06	1.73440E-06	1.73440E-06	1.92092E-06
**F22**	N/A	1.73440E-06	1.73331E-06	1.73440E-06	1.73440E-06	1.73440E-06	1.73440E-06
**F23**	N/A	1.73440E-06	1.73440E-06	1.73440E-06	1.73440E-06	1.73440E-06	1.92092E-06
**F24**	N/A	1.73440E-06	2.87860E-06	1.73440E-06	1.73440E-06	1.73440E-06	3.51524E-06
**F25**	N/A	1.73440E-06	2.18267E-02	1.73440E-06	1.73440E-06	1.73440E-06	1.73440E-06
**F26**	N/A	1.73440E-06	1.73440E-06	1.73440E-06	1.73440E-06	1.73440E-06	2.12664E-06
**F27**	N/A	1.73440E-06	6.56411E-02	1.73440E-06	1.73440E-06	1.73440E-06	2.16302E-05
**F28**	N/A	1.73440E-06	3.11232E-05	1.73440E-06	1.73440E-06	1.92092E-06	1.73440E-06
**F29**	N/A	1.73440E-06	2.16302E-05	1.73440E-06	1.73440E-06	1.73440E-06	1.70877E-03
**F30**	N/A	1.73440E-06	1.73440E-06	1.73440E-06	1.73440E-06	3.51524E-06	1.73440E-06
**Rank**	**1**	9	6	7	11	2	4

**Table 7:** The p-values of algorithm.

Function	MGFOA	CCMWOA	QCSCA	BWOA	CCMSCSA	GCHHO
**F1**	N/A	1.73440E-06	1.65655E-02	1.73440E-06	1.73440E-06	1.73440E-06
**F2**	N/A	1.73440E-06	1.73440E-06	1.73440E-06	4.28569E-06	1.73440E-06
**F3**	N/A	1.73440E-06	1.73440E-06	1.73440E-06	1.73440E-06	2.87860E-06
**F4**	N/A	1.73440E-06	5.31968E-03	1.92092E-06	2.13358E-01	1.73440E-06
**F5**	N/A	1.73440E-06	1.73440E-06	1.92092E-06	1.73440E-06	1.73440E-06
**F6**	N/A	1.73440E-06	1.73440E-06	1.73440E-06	1.73440E-06	1.73440E-06
**F7**	N/A	1.73440E-06	1.23808E-05	8.46608E-06	1.73440E-06	1.73440E-06
**F8**	N/A	1.73440E-06	1.73440E-06	2.12664E-06	1.73440E-06	1.73440E-06
**F9**	N/A	1.73440E-06	1.73440E-06	1.73440E-06	1.73440E-06	1.73440E-06
**F10**	N/A	1.05695E-04	4.65283E-01	9.36756E-02	3.88111E-04	8.72967E-03
**F11**	N/A	1.73440E-06	3.51524E-06	1.73440E-06	1.73440E-06	1.73440E-06
**F12**	N/A	1.73440E-06	2.62299E-01	1.73440E-06	1.73440E-06	1.73440E-06
**F13**	N/A	1.73440E-06	8.93644E-01	1.73440E-06	1.73440E-06	2.35342E-06
**F14**	N/A	4.28569E-06	1.17481E-02	1.84622E-01	5.21649E-06	7.34325E-01
**F15**	N/A	2.12664E-06	8.72967E-03	3.40526E-05	1.73440E-06	6.98378E-06
**F16**	N/A	1.92092E-06	2.89477E-01	2.53644E-01	1.02463E-05	6.33914E-06
**F17**	N/A	3.51524E-06	3.60943E-03	5.03833E-01	1.97295E-05	7.69086E-06
**F18**	N/A	2.12664E-06	8.93644E-01	1.31942E-02	4.65283E-01	2.76527E-03
**F19**	N/A	1.73440E-06	3.37885E-03	5.21649E-06	1.73440E-06	9.31566E-06
**F20**	N/A	7.69086E-06	8.61213E-01	8.45080E-01	5.79245E-05	6.98378E-06
**F21**	N/A	1.73440E-06	1.73440E-06	1.73440E-06	1.73440E-06	1.73440E-06
**F22**	N/A	1.73440E-06	8.29810E-06	1.73440E-06	1.73440E-06	1.73440E-06
**F23**	N/A	1.73440E-06	1.92092E-06	4.72920E-06	1.92092E-06	1.73440E-06
**F24**	N/A	1.73440E-06	1.36011E-05	2.41470E-03	1.73440E-06	2.60333E-06
**F25**	N/A	1.73440E-06	1.35948E-04	1.73440E-06	1.24526E-02	1.73440E-06
**F26**	N/A	1.73440E-06	1.73440E-06	3.18168E-06	7.27105E-03	1.73440E-06
**F27**	N/A	1.73440E-06	5.28725E-04	9.62659E-04	3.58884E-04	1.83258E-03
**F28**	N/A	1.73440E-06	9.71105E-05	1.73440E-06	1.25057E-04	1.73440E-06
**F29**	N/A	1.92092E-06	1.38204E-03	3.68261E-02	1.92092E-06	3.18168E-06
**F30**	N/A	1.73440E-06	6.63921E-04	1.73440E-06	1.73440E-06	1.92092E-06
**Rank**	**1**	12	3	10	8	5

**Fig 6:**
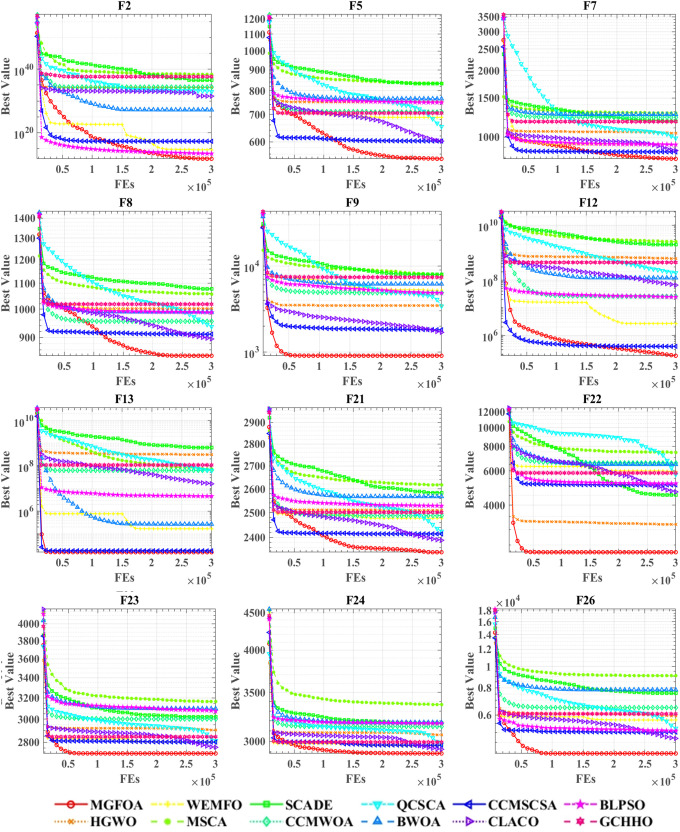
Performance comparisons of MGFOA with state-of-the-art competitors on the IEEE CEC 2017.

Fig 6 demonstrates the convergence curves of MGFOA and its counterparts on the CEC 2017 benchmark functions. Convergence curves are fundamental in evolutionary algorithm research, providing a visual summary of the algorithm’s behavior during optimization. These plots delineate the search process’s progress within the solution space, highlighting convergence speed, stability, and potential issues like premature convergence or oscillation. By analyzing these curves, researchers can adjust algorithm parameters for enhanced performance, aligning the algorithm more closely with specific problem requirements. Thus, convergence curves are crucial for evaluating algorithmic performance and refining designs in evolutionary computing. The graph illustrates convergence curves for all algorithms tested across twelve benchmark functions, with the x-axis representing iteration count and the y-axis representing optimization values. For functions F5, F8, F22, and F26, MGFOA exhibits superior convergence performance, rapidly reaching and holding the lowest optimization values. In other graphs, even in complex scenarios with closely packed convergence curves, MGFOA consistently outperforms its competitors in optimization value.

#### 4.5.2 Comparative experiments at CEC 2022 benchmark functions.

The competing algorithms involved in this experiment include HGWO [44], WEMFO [45], mSCA [46], SCADE [47], CCMWOA [48], QCSCA [49], BWOA [50], CCMSCSA [51], CLACO [52], BLPSO [53], GCHHO [54]. Table 8 presents a detailed comparison of MGFOA with alternative competing algorithms using the IEEE CEC 2022 benchmark functions. This analysis includes each algorithm’s rank, performance against MGFOA indicated by wins/draws/losses (+/ = /-), and the average performance score (AVG) across multiple runs. “+” indicates that MGFOA outperforms the optimizer, “-” means MGFOA underperforms compared to the optimizer, and “=” denotes no significant difference in performance between MGFOA and the optimizer. The Wilcoxon signed-rank test [42] and Friedman test [43] was employed to evaluate performance. MGFOA secures the top rank with an impressive average score of 2.18E + 00, denoted by the “~” symbol in the + / = /- column, indicating that MGFOA serves as the benchmark algorithm in this comparative study. This underscores MGFOA’s robust optimization capabilities and its consistent ability to achieve optimal solutions across the diverse set of benchmark functions in IEEE CEC 2022. Among the competing algorithms, QCSCA secures the 2nd rank with an average score of 2.89E + 00. Despite MGFOA’s top rank, QCSCA demonstrates competitive performance with a 6/0/6 + / = /- metric, indicating instances where it outperforms MGFOA and others where it falls short. HGWO, BWOA, and WEMFO, ranked 9th, 8th, and 6th respectively, exhibit average scores of 7.13E + 00, 7.21E + 00, and 5.15E + 00. Their performance metrics (10/1/1, 9/2/1, and 9/2/1 + / = /- respectively) indicate competitive instances against MGFOA but with higher average scores, suggesting less consistent performance compared to MGFOA. Algorithms such as CLACO and GCHHO, ranked 3rd and 4th respectively, present robust competition with average scores of 3.43E + 00 and 4.21E + 00. CLACO’s 4/2/6 + / = /- metric suggests mixed performance against MGFOA, while GCHHO’s 7/2/3 + / = /- metric indicates competitive performance with instances of outperformance. On the other hand, algorithms like mSCA, SCADE, CCMWOA, and CCMSCSA (Chaos-Crow Search with Multiverse Strategy Algorithm), ranked 7th, 12th, 11th, and 10th respectively. Their performance metrics indicate varying degrees of competitiveness against MGFOA, with instances of success and failure in optimization tasks. In summary, the experimental results demonstrate that MGFOA outperforms many alternative algorithms on the IEEE CEC 2022 benchmark functions, highlighting its robustness and effectiveness in global optimization tasks. The findings establish MGFOA as a leading algorithm in the field, with significant potential for practical applications across diverse domains.

**Table 8:** Experiments comparing MGFOA with alternative competing algorithms at IEEE CEC 2022 benchmark functions.

Algorithm	Rank	+/ = /-	AVG
MGFOA	1	~	2.18E + 00
HGWO	9	10/1/1	7.13E + 00
SCADE	12	10/2/0	8.43E + 00
CCMWOA	11	9/3/0	8.21E + 00
QCSCA	2	6/0/6	2.89E + 00
BWOA	8	9/2/1	7.21E + 00
WEMFO	6	9/2/1	5.15E + 00
mSCA	7	10/0/2	5.76E + 00
CCMSCSA	10	10/1/1	8.90E + 00
CLACO	3	4/2/6	3.43E + 00
BLPSO	5	8/2/2	4.98E + 00
GCHHO	4	7/2/3	4.21E + 00

**Fig 7:**
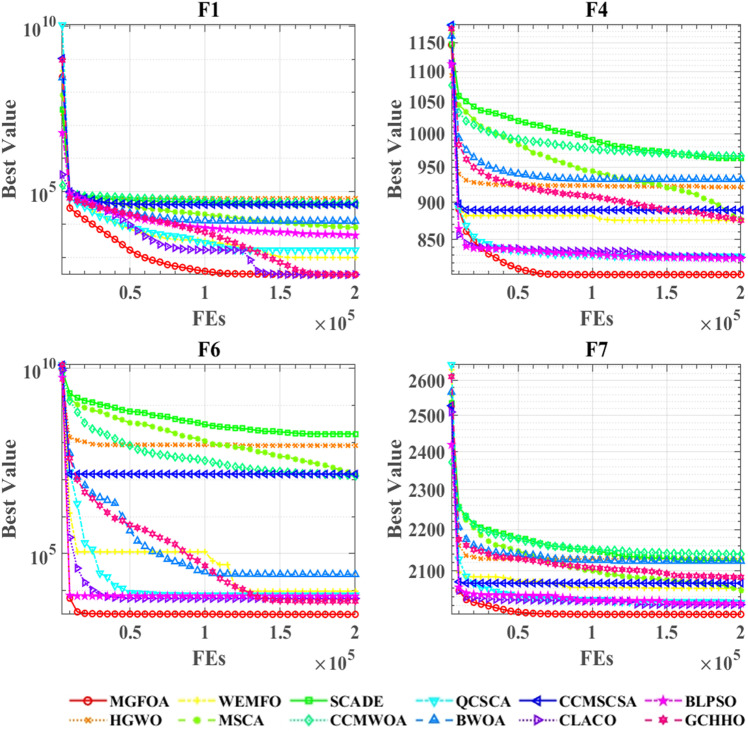
Performance comparisons of MGFOA with state-of-the-art competitors on the IEEE CEC 2022 benchmark functions.

Fig 7 illustrates the convergence curves for MGFOA and its competitors on the CEC 2022 benchmark functions. The diagram highlights the convergence paths for all compared algorithms across nine test functions, with the horizontal axis depicting the number of iterations and the vertical axis indicating the optimization value. For functions F1, F4, F6, and F7, MGFOA exhibits significant convergence benefits, quickly achieving optimal values and reaching the lowest optimization levels. Even in other more complex scenarios where convergence curves are densely clustered, MGFOA consistently reaches the best optimization results.

## 5 Application to oil reservoir production

The primary aim in optimizing reservoir production is to determine the optimal solutions for each well to maximize the net present value (NPV). Nevertheless, with the increase in the number of wells and production cycles, the solution space expands combinatorially, resulting in a higher dimensionality of optimization variables. As a result, this challenge is classified as a typical NP-hard problem, making it an ideal scenario for utilizing evolutionary algorithms.

Within this section, MGFOA is employed in the context of a three-channel reservoir model using the numerical simulation software Eclipse. Following this, we assess MGFOA’s performance by benchmarking it against several traditional evolutionary algorithms. In our experimental design, we reduce complexity by disregarding the nonlinear constraints commonly found in oilfield production. Instead, the emphasis is placed on optimizing the objective function, specifically the NPV, as specified in equation 15.


$$NPV(x,z)=\sum_{t=1}^{n}\;\Delta t\frac{Q_{o,t}\cdot r_{o}-Q_{w,t}\cdot r_{w}-Q_{i,t}\cdot r_{i}}{(1+b{)}^{p_{t}}}$$ (15)

In this context, x denotes the set of variables subject to optimization, which in this experiment corresponds to the injection and recovery rates of each well. The variable z is utilized as the state parameter of the model, describing the numerical reservoir model’s construction. The total simulation duration is represented by n, with $Q_{o,t}$, $Q_{w,t}$, $Q_{i,t}$ indicating the oil production rate, water production rate, and water injection rate, respectively, at time t. The variable $r_{o}$ signifies oil revenue, while $r_{w}$ and $r_{i}$ represent the costs of water treatment and injection, respectively. The term b stands for the average annual interest rate, and $p_{t}$ denotes the number of elapsed years.

**Fig 8:**
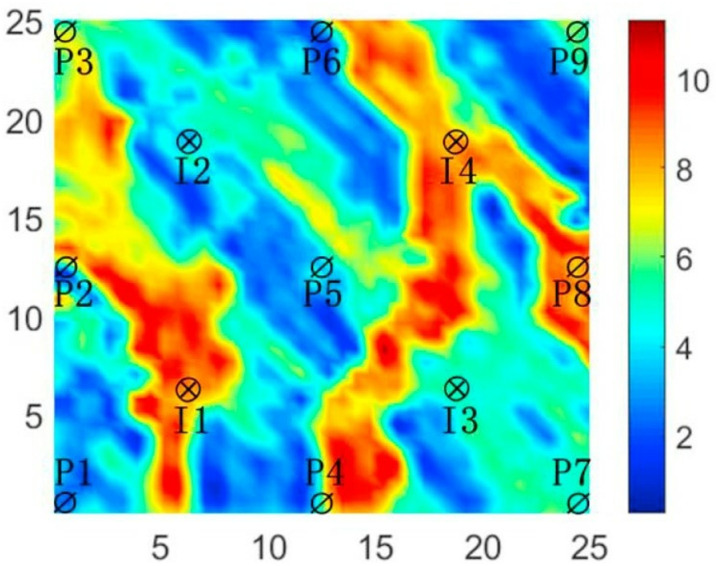
Log-permeability distribution of three-channel model.

The three-channel reservoir model is described as a non-uniform, two-dimensional reservoir, comprising nine production wells and four injection wells arranged in a 5-point configuration. The model is represented by a 25 * 25 * 1 grid, where each cell measures 100 feet in length. Each cell has a thickness of 20 feet, and the porosity is consistently set at 0.2. Fig 8 depicts the detailed distribution of the modeled permeability.

In this production optimization scenario, the optimization variables include the injection rates for each injection well and the fluid recovery rates for the production wells. The water injection rate is between 0 and 500 STB/DAY, while the water extraction rate for production wells ranges from 0 to 200 STB/DAY. The thermal storage is utilized over 1800 days, and the decision time step is 360 days, resulting in a decision variable dimensionality of 65. The fitness function used for optimization is the NPV, with the oil price fixed at 80.0 USD/STB, and the costs for water injection and treatment are both set at 5.0 USD/STB. For simplicity, the model assumes a zero average annual interest rate.

**Fig 9:**
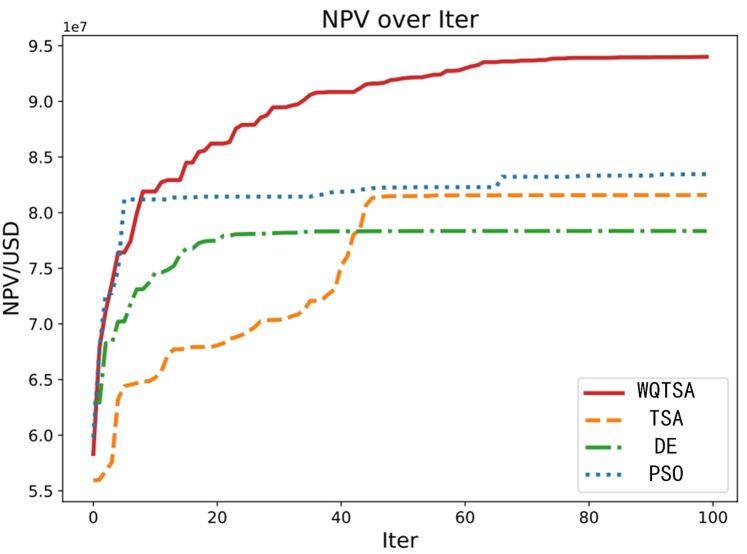
NPV obtained by the algorithms with iteration.

To validate the effectiveness of the algorithm enhancement, MGFOA was optimized in parallel with FOA, GWO, SMA, MFO, and WOA for model comparison. To ensure experimental fairness, each optimization was executed 10 times, and the average of the final NPV values was considered for analysis. Fig 9 depicts the optimal NPV values obtained by the four methods as a function of the number of iterations. The figure clearly shows that MGFOA consistently achieves higher NPV values than FOA, GWO, SMA, MFO, and WOA within the same number of iterations.

**Fig 10:**
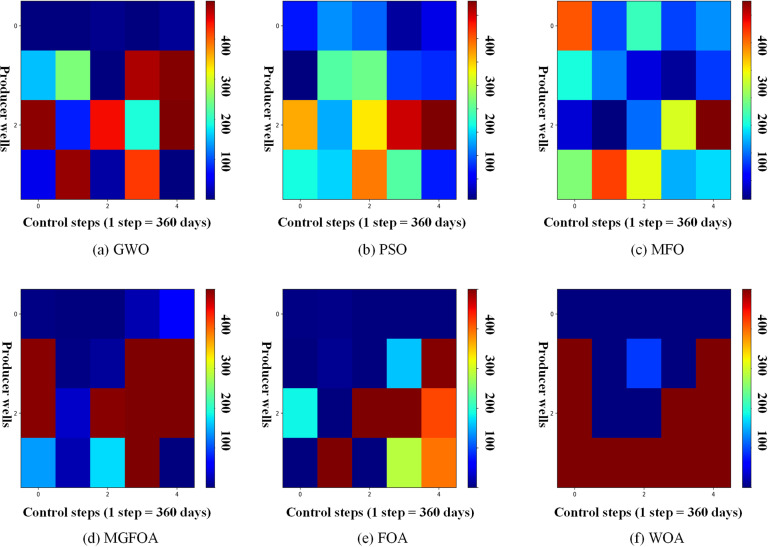
The optimal water-injection rate obtained by each algorithm for the three-channel model.

**Fig 11:**
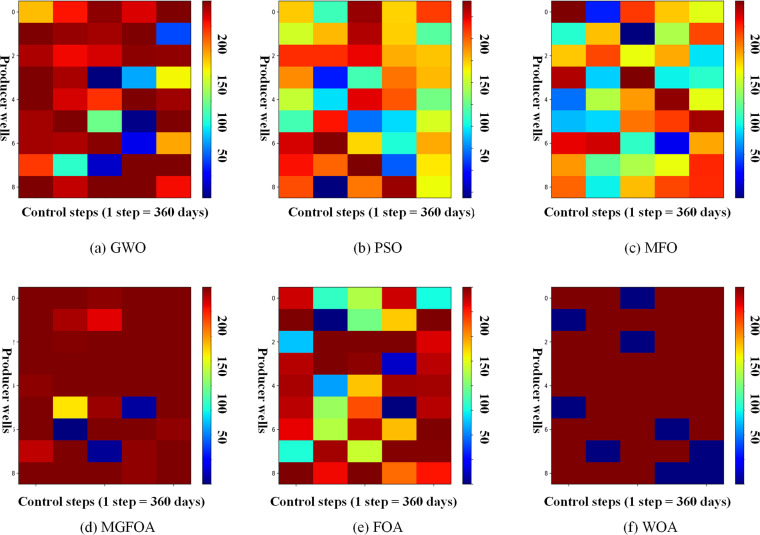
The optimal liquid-production rate obtained by each algorithm for the three-channel model.

Figs 10 and 11 depict the final optimization schemes for water injection rates and liquid production rates for the six methods. In these figures, the horizontal axis indicates the practice step size, and the vertical axis denotes the well number. Fig 10 showcases the FOAinjection well regulation scheme, which highlights significant fluctuations in injection rates for the same wells at consecutive control step values. This lack of stability is problematic for field applications, as variable injection rates can negatively impact bottomhole pressure, potentially harming the reservoir and hindering sustainable development. Conversely, MGFOA offers a more stable production scheme compared to FOA, as shown in the figures. Fig 11 illustrates the liquid production rates for the six algorithms, demonstrating that MGFOA has a clear advantage over the other five algorithms.

## 6 Conclusions and future works

This paper presents a novel evolutionary algorithm named MGFOA, which is an enhancement of the FOA incorporating a chaotic exploitation mechanism and an orthogonal learning strategy. The chaotic exploitation mechanism enhances MGFOA’s global search efficiency, preventing it from settling into local optima. The orthogonal learning strategy strengthens its local search capability, ensuring that the optimal solution is not missed. To verify the optimization performance of MGFOA, a series of comprehensive experiments were conducted. Ablation studies at IEEE CEC 2017 verified the effectiveness of the two mechanisms in MGFOA. By selectively disabling one mechanism, it was shown that both mechanisms improve COFOA’s performance. Scalability tests demonstrated MGFOA’s efficacy in handling large-scale, multi-dimensional optimization problems. Search trace analyses illustrated MGFOA’s search trajectory, highlighting that population members consistently search near the global optimum. Comparative experiments with other state-of-the-art algorithms highlighted MGFOA’s superior optimization capabilities. Lastly, MGFOA was applied to oil and gas production optimization, confirming its excellent performance in real-world scenarios.

The advantage of this algorithm lies in its excellent global optimization capability and does not consume too much computing resources. Meanwhile, we note that although the proposed algorithm performs well in oil and gas production optimization, we have not validated its optimization capabilities in other fields. In future research, we will continue to explore the optimization capabilities of this algorithm in other fields and continuously improve its optimization performance. Looking ahead, the FOA can be enhanced with more efficient mechanisms to improve its already established performance. This algorithm can then be extended to various fields such as feature selection, extreme learning machines, and engineering optimization challenges. Moreover, creating a binary version of the algorithm could make it suitable for tackling discrete optimization problems. The limitation of the algorithm proposed in this paper is that the performance of the algorithm is not tested in a wider range of applications. In our future work, we will explore more areas of optimization, such as optimization for cloud resource scheduling and deep learning.

## References

[1] Wiggins M, Startzman R, editors. An approach to reservoir management. SPE Annual Technical Conference and Exhibition? SPE; 1990.

[2] Lake L, Johns R, Rossen W, Pope G. Fundamentals of Enhanced Oil Recovery. 2014.

[3] Rao SS. Engineering optimization: theory and practice. John Wiley & Sons; 2019.

[4] Liu X, Reynolds AC, editors. Gradient-based multiobjective optimization with applications to waterflooding optimization. ECMOR XIV-14th European Conference on the Mathematics of Oil Recovery; 2014: European Association of Geoscientists & Engineers.

[5] Al-Aghbari M, Al-Wadhahi M, Gujarathi AM. Multi-objective optimization of Brugge field for short-term and long-term waterflood management. Arabian Journal for Science and Engineering. 2022:1–19.

[6] Zhang X, Wang D, Zhou Z, Ma Y. Robust Low-Rank Tensor Recovery with Rectification and Alignment. IEEE Transactions on Pattern Analysis and Machine Intelligence. 2019;10.1109/TPAMI.2019.2929043:1. doi: 10.1109/TPAMI.2019.2929043 31329109

[7] Chen H, Xu Y, Wang M, Zhao X. A Balanced Whale Optimization Algorithm for Constrained Engineering Design Problems. Applied Mathematical Modelling. 2019. doi: 10.1016/j.apm.2019.02.004

[8] Zhao X, Zhang X, Cai Z, Tian X, Wang X, Huang Y, et al. Chaos enhanced grey wolf optimization wrapped ELM for diagnosis of paraquat-poisoned patients. Comput Biol Chem. 2019;78:481–90. doi: 10.1016/j.compbiolchem.2018.11.017 30501982

[9] Zhang Q, Chen H, Luo J, Xu Y, Wu C, Li C. Chaos enhanced bacterial foraging optimization for global optimization. IEEE Access. 2018;6:64905–19. doi: 10.1109/ACCESS.2018.2876996

[10] Luo J, Chen H, Zhang Q, Xu Y, Huang H, Zhao X. An improved grasshopper optimization algorithm with application to financial stress prediction. Applied Mathematical Modelling. 2018;64:654–68. doi: 10.1016/j.apm.2018.07.044

[11] Shen L, Chen H, Yu Z, Kang W, Zhang B, Li H, et al. Evolving support vector machines using fruit fly optimization for medical data classification. Knowledge-Based Systems. 2016;96:61–75. doi: 10.1016/j.knosys.2016.01.002

[12] Deng W, Zhao H, Zou L, Li G, Yang X, Wu D. A novel collaborative optimization algorithm in solving complex optimization problems. Soft Computing. 2017;21(15):4387–98. doi: 10.1007/s00500-016-2071-8

[13] Deng W, Zhao H, Yang X, Xiong J, Sun M, Li B. Study on an improved adaptive PSO algorithm for solving multi-objective gate assignment. Applied Soft Computing. 2017;59:288–302. doi: 10.1016/j.asoc.2017.06.004

[14] Deng W, Xu J, Zhao H. An Improved Ant Colony Optimization Algorithm Based on Hybrid Strategies for Scheduling Problem. IEEE Access. 2019;7:20281–92. doi: 10.1109/ACCESS.2019.2897580

[15] Yu H, Zhao N, Wang P, Chen H, Li C. Chaos-enhanced synchronized bat optimizer. Applied Mathematical Modelling. 2019. doi: 10.1016/j.apm.2019.09.029

[16] Zhang X, Xu Y, Yu C, Heidari AA, Li S, Chen H, et al. Gaussian mutational chaotic fruit fly-built optimization and feature selection. Expert Systems with Applications. 2019:112976. doi: 10.1016/j.eswa.2019.112976

[17] Zhang X, Hu W, Qu W, Maybank S. Multiple Object Tracking Via Species-Based Particle Swarm Optimization. IEEE Trans Circuits Syst Video Technol. 2010;20(11):1590–602. doi: 10.1109/tcsvt.2010.2087455

[18] Yang XS. Firefly algorithms for multimodal optimization. Lecture Notes in Computer Science (including subseries Lecture Notes in Artificial Intelligence and Lecture Notes in Bioinformatics). 2009. p. 169–78.

[19] Mirjalili S. Moth-flame optimization algorithm: A novel nature-inspired heuristic paradigm. Knowledge-Based Systems. 2015;89:228–49. doi: 10.1016/j.knosys.2015.07.006

[20] Kennedy J, Eberhart R, editors. Particle swarm optimization. IEEE International Conference on Neural Networks - Conference Proceedings; 1995.

[21] Yang XS. A new metaheuristic Bat-inspired Algorithm. Studies in Computational Intelligence. 2010. p. 65–74.

[22] Mirjalili S, Lewis A. The Whale Optimization Algorithm. Advances in Engineering Software. 2016;95:51–67. doi: 10.1016/j.advengsoft.2016.01.008

[23] Heidari AA, Mirjalili S, Faris H, Aljarah I, Mafarja M, Chen H. Harris hawks optimization: Algorithm and applications. Future Generation Computer Systems. 2019;97:849–72. doi: 10.1016/j.future.2019.02.028

[24] Pan WT. A new Fruit Fly Optimization Algorithm: Taking the financial distress model as an example. Knowledge-Based Systems. 2012;26:69–74. doi: 10.1016/j.knosys.2011.07.001

[25] Wolpert DH, Macready WG. No free lunch theorems for optimization. IEEE Trans Evol Computat. 1997;1(1):67–82. doi: 10.1109/4235.585893

[26] Wang Z-Z, Zhang K, Chen G-D, Zhang J-D, Wang W-D, Wang H-C, et al. Evolutionary-assisted reinforcement learning for reservoir real-time production optimization under uncertainty. Petroleum Science. 2023;20(1):261–76. doi: 10.1016/j.petsci.2022.08.016

[27] Du S-Y, Zhao X-G, Xie C-Y, Zhu J-W, Wang J-L, Yang J-S, et al. Data-driven production optimization using particle swarm algorithm based on the ensemble-learning proxy model. Petroleum Science. 2023;20(5):2951–66. doi: 10.1016/j.petsci.2023.04.001

[28] Ng CSW, Jahanbani Ghahfarokhi A, Nait Amar M. Production optimization under waterflooding with long short-term memory and metaheuristic algorithm. Petroleum. 2023;9(1):53–60. doi: 10.1016/j.petlm.2021.12.008

[29] Alawad NA, Abed-alguni BH, Saleh II. Improved arithmetic optimization algorithm for patient admission scheduling problem. Soft Computing. 2024;28(7):5853–79.

[30] Abed-alguni BH. Island-based cuckoo search with highly disruptive polynomial mutation. International Journal of Artificial Intelligence. 2019;17(1):57–82.

[31] Abed-alguni BH, Paul D. Island-based cuckoo search with elite opposition-based learning and multiple mutation methods for solving discrete and continuous optimization problems. 2021.

[32] Abed-Alguni BH, Alawad NA. Distributed Grey Wolf Optimizer for scheduling of workflow applications in cloud environments. Applied Soft Computing. 2021;102:107113. doi: 10.1016/j.asoc.2021.107113

[33] Nadimi-Shahraki MH, Taghian S, Mirjalili S. An improved grey wolf optimizer for solving engineering problems. Expert Systems with Applications. 2021;166:113917. doi: 10.1016/j.eswa.2020.113917

[34] Nadimi-Shahraki MH, Taghian S, Mirjalili S, Faris H. MTDE: An effective multi-trial vector-based differential evolution algorithm and its applications for engineering design problems. Applied Soft Computing. 2020;97:106761. doi: 10.1016/j.asoc.2020.106761

[35] Nadimi-Shahraki MH, Taghian S, Javaheri D, Sadiq AS, Khodadadi N, Mirjalili S. MTV-SCA: multi-trial vector-based sine cosine algorithm. Cluster Computing. 2024:1–45.

[36] Nadimi-Shahraki MH, Taghian S, Mirjalili S, Abualigah L, Abd Elaziz M, Oliva D. EWOA-OPF: Effective whale optimization algorithm to solve optimal power flow problem. Electronics. 2021;10(23):2975. doi: 10.3390/electronics10232975

[37] Nadimi-Shahraki MH, Moeini E, Taghian S, Mirjalili S. Discrete Improved Grey Wolf Optimizer for Community Detection. Journal of Bionic Engineering. 2023;20(5):2331–58. doi: 10.1007/s42235-023-00387-1

[38] Tabatabaei S. An energy-aware protocol in wireless sensor networks using the scattered search algorithm and fuzzy logic. PLoS One. 2024;19(11):e0297728. doi: 10.1371/journal.pone.0297728 39495811 PMC11534263

[39] Tabatabaei S. Introducing a new routing method in the MANET using the symbionts search algorithm. PLoS One. 2023;18(8):e0290091. doi: 10.1371/journal.pone.0290091 37624812 PMC10456139

[40] Tabatabaei S. Provide energy-aware routing protocol in wireless sensor networks using bacterial foraging optimization algorithm and mobile sink. PLoS One. 2022;17(3):e0265113. doi: 10.1371/journal.pone.0265113 35320290 PMC8942282

[41] Tabatabaei S. New Energy Efficient Management Approach for Wireless Sensor Networks in Target Tracking Using Vortex Search Algorithm. Heliyon. 2025.

[42] Demsar J. Statistical comparisons of classifiers over multiple data sets. Journal of Machine Learning Research. 2006;7:1–30.

[43] García S, Fernández A, Luengo J, Herrera F. Advanced nonparametric tests for multiple comparisons in the design of experiments in computational intelligence and data mining: Experimental analysis of power. Information Sciences. 2010;180(10):2044–64. doi: 10.1016/j.ins.2009.12.010

[44] Deng S, Wang X, Zhu Y, Lv F, Wang J. Hybrid Grey Wolf Optimization Algorithm–Based Support Vector Machine for Groutability Prediction of Fractured Rock Mass. Journal of Computing in Civil Engineering. 2019;33(2):04018065. doi: 10.1061/(ASCE)CP.1943-5487.0000814

[45] Shan W, Qiao Z, Heidari AA, Chen H, Turabieh H, Teng Y. Double adaptive weights for stabilization of moth flame optimizer: Balance analysis, engineering cases, and medical diagnosis. Knowledge-Based Systems. 2021;214:106728. doi: 10.1016/j.knosys.2020.106728

[46] Gupta S, Deep K. A hybrid self-adaptive sine cosine algorithm with opposition based learning. Expert Systems with Applications. 2019;119:210–30. doi: 10.1016/j.eswa.2018.10.050

[47] Alambeigi F, Aghajani S, Pedram SA, Speyer JL, Rosen J, Iordachita I, Taylor RH, et al. SCADE: Simultaneous Sensor Calibration and Deformation Estimation of FBG-Equipped Unmodeled Continuum Manipulators. IEEE Trans Robot. 2020;36(1):222–39. doi: 10.1109/TRO.2019.2946726 32661460 PMC7357879

[48] Luo J, Chen H, Heidari AA, Xu Y, Zhang Q, Li C. Multi-strategy boosted mutative whale-inspired optimization approaches. Applied Mathematical Modelling. 2019;73:109–23. doi: 10.1016/j.apm.2019.03.046

[49] Hu H, Shan W, Tang Y, Heidari AA, Chen H, Liu H, et al. Horizontal and vertical crossover of sine cosine algorithm with quick moves for optimization and feature selection. Journal of Computational Design and Engineering. 2022;9(6):2524–55. doi: 10.1093/jcde/qwac119

[50] Reddy KS, Panwar L, Panigrahi BK, Kumar R. Binary whale optimization algorithm: a new metaheuristic approach for profit-based unit commitment problems in competitive electricity markets. Engineering Optimization. 2019;51(3):369–89. doi: 10.1080/0305215X.2018.1463527

[51] Shan W, Hu H, Cai Z, Chen H, Liu H, Wang M, et al. Multi-strategies boosted mutative crow search algorithm for global tasks: Cases of continuous and discrete optimization. Journal of Bionic Engineering. 2022;19(6):1830–49. doi: 10.1007/s42235-022-00228-7

[52] Liu L, Zhao D, Yu F, Heidari AA, Li C, Ouyang J, et al. Ant colony optimization with Cauchy and greedy Levy mutations for multilevel COVID 19 X-ray image segmentation. Comput Biol Med. 2021;136:104609. doi: 10.1016/j.compbiomed.2021.104609 34293587 PMC8254401

[53] Chen X, Li K, Xu B, Yang Z. Biogeography-based learning particle swarm optimization for combined heat and power economic dispatch problem. Knowledge-Based Systems. 2020;208:106463. doi: 10.1016/j.knosys.2020.106463

[54] Song S, Wang P, Heidari AA, Wang M, Zhao X, Chen H, et al. Dimension decided Harris hawks optimization with Gaussian mutation: Balance analysis and diversity patterns. Knowledge-Based Systems. 2021;215:106425. doi: 10.1016/j.knosys.2020.106425

